# Liquid metal-filled magnetorheological elastomer with positive piezoconductivity

**DOI:** 10.1038/s41467-019-09325-4

**Published:** 2019-03-21

**Authors:** Guolin Yun, Shi-Yang Tang, Shuaishuai Sun, Dan Yuan, Qianbin Zhao, Lei Deng, Sheng Yan, Haiping Du, Michael D. Dickey, Weihua Li

**Affiliations:** 1 School of Mechanical, Materials, Mechatronic and Biomedical Engineering, University of Wollongong, Wollongong, NSW 2522 Australia; 2 Department of Chemistry, The University of Tokyo, Tokyo, 113-8654 Japan; 3 School of Electrical, Computer and Telecommunications Engineering, University of Wollongong, Wollongong, NSW 2522 Australia; 4 Department of Chemical and Biomolecular Engineering, North Carolina State University, Raleigh, NC 27695 USA

## Abstract

Conductive elastic composites have been used widely in soft electronics and soft robotics. These composites are typically a mixture of conductive fillers within elastomeric substrates. They can sense strain via changes in resistance resulting from separation of the fillers during elongation. Thus, most elastic composites exhibit a negative piezoconductive effect, i.e. the conductivity decreases under tensile strain. This property is undesirable for stretchable conductors since such composites may become less conductive during deformation. Here, we report a liquid metal-filled magnetorheological elastomer comprising a hybrid of fillers of liquid metal microdroplets and metallic magnetic microparticles. The composite’s resistivity reaches a maximum value in the relaxed state and drops drastically under any deformation, indicating that the composite exhibits an unconventional positive piezoconductive effect. We further investigate the magnetic field-responsive thermal properties of the composite and demonstrate several proof-of-concept applications. This composite has prospective applications in sensors, stretchable conductors, and responsive thermal interfaces.

## Abstract (web summary)

Liquid metal-filled elastic composites for strain sensing devices exhibit reduced conductivity under strain, which limits their usefulness. Here, the authors report a positive piezoconductive effect in liquid metal-filled magnetorheological elastomers and illustrate proof-of concept applications.

## Introduction

With the rapid development of soft electronics, attention to conductive elastic composites is rising. These composites have a variety of applications such as sensors, wearables, and robots^1–4^. Most of the composite materials consist of conductive fillers dispersed within elastomeric substrates^5^. The physical properties, shape, and concentration of the fillers affects the thermal and electrical conductivities of the composite^6–8^. The fillers also influence the sensitivity of the composites to physical conditions, such as strain, pressure, magnetic field, and temperature^9^. The elastic matrix, which is normally a dielectric insulator, influences the mechanical properties of the composite^10^.

Traditionally, conductive solid metals or carbon-based materials have been used as fillers for elastic conductors. Composites that contain solid metal fillers have a range of excellent properties, such as low density, low cost, and easy processing^11^. Particle fillers separate from each other upon stretching the composite, causing the electrical conductivity of the material to drop drastically. Metal flakes and wires can overlap in the composite to form conductive networks, as such, these composites often have high conductivity under stretching, but their gauge factors are generally low^12–14^. Similarly, carbon-based materials including carbon black, carbon fibre, carbon nanotubes and graphene have been used as fillers. Composites with carbon fillers possess better oxidation resistance and higher mechanical strength in comparison with materials with solid metal fillers^15,16^. Nonetheless, the electrical conductivity of these composites is generally low and less sensitive to mechanical deformation^17^.

Recently, gallium-based liquid metal alloys have also been employed as a conductive filler^18,19^. EGaIn, a mixture of 75% gallium and 25% indium is commonly used due to its desirable properties, such as high electrical/thermal conductivities and high deformability^3,20^. Liquid metal tends to break into microdroplets in the elastomer upon mixing. During deformation, the microdroplets can deform along with the matrix and retain its high conductivity at high strains, which can be used in soft robotics^21^. Liquid metal can also enhance the toughness of composites because it can divert the propagation of tears^22^. However, composites using only liquid metal are either insulators or conductors, which makes their change of resistance relatively insensitive to strain, limiting their application as sensors^3,23^. To solve this problem, some recent studies added EGaIn together with other conductive fillers to form hybrid composites^24^. EGaIn can form conductive pathways between adjacent conductive particles and improve the material conductivity under stretching. It can also improve the stability of the electrical properties during cyclic stretching and the tensile limit of the composite^24^.

Regardless of the material selection, the electrical contact between the conductive fillers in most composites determines the conductivity. During stretching, conductive fillers separate from each other and the number of electrical contacts decreases, leading to a significant increase in resistance. Thus, most composites exhibit a negative piezoconductive effect, although recent studies suggest liquid metal-filled composites can minimise the piezoconductive effect during stretching^24–27,35^. Elastic composites with a positive piezoconductive property, together with the characteristic of high sensitivity to strain, are underexplored.

In this work, we report a liquid metal-filled magnetorheological elastomer (LMMRE) comprising EGaIn microdroplets and metallic magnetic microparticles. Compared with conventional composites, this LMMRE uniquely reaches a maximum resistance in the relaxed state and its resistivity can drop sharply under any mechanical deformation, including compression, stretching, bending, and twisting. This indicates that the LMMRE can exhibit an unconventional positive piezoconductive effect. We thoroughly explored the effects of particle (size, content, type, and morphology), elastomer, liquid metal content, and curing process on the physical properties of the LMMRE. In addition, we further studied the magnetic and thermal properties of the LMMRE, and demonstrated several proof-of-concept applications such as forming field-responsive electronic components and constructing intelligent heating devices based on the unique properties of this material.

## Results

### Production of the LMMRE

**Fig. 1:**
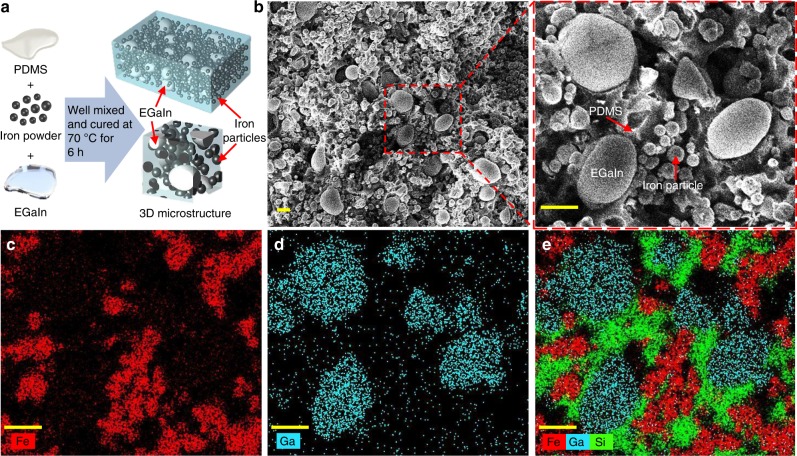
Production of the liquid metal-filled magnetorheological elastomer (LMMRE). **a** Schematic of the procedure for fabricating the LMMRE. **b** Scanning electron microscopy (SEM) images of the obtained LMMRE. **c**–**e** Energy dispersive X-ray spectroscopy (EDS) element mappings of the LMMRE. Scale bars are 10 µm

Figure 1a illustrates the fabrication of LMMRE’s and a schematic of its microstructure. Briefly, we mixed and subsequently cured polydimethylsiloxane (PDMS), ferromagnetic microparticles composed of carbonyl iron (Fe) (2–5 µm diameter), and EGaIn. Figure 1b shows the scanning electron microscopy (SEM) images of the LMMRE produced using PDMS (1 g, 58.5 v%, curing agent/PDMS ratio of 1:7), carbonyl Fe microparticles (4 g, 29.8 v%), and EGaIn (1.25 g, 11.7 v%). A micrograph of a LMMRE and its cross section are shown in Supplementary Fig. 1. EGaIn breaks into microdroplets (4–30 µm diameter) during mixing, as evident in the SEM image of the composite. These materials distribute uniformly within the composite. Figure 1c–e show the energy dispersive X-ray spectroscopy (EDS) analysis of each material for the red-dashed area given in Fig. 1b. The distribution of Fe particles, EGaIn, and PDMS was analysed by mapping their characteristic elements. Since the EDS analysis only scans the elements on the surface layer of the material, the PDMS matrix is obscured by Fe particles, EGaIn droplets, and their shadows, resulting in a seemingly discontinuous distribution of silicon (Si) elements from the PDMS. As can be seen from the SEM images, the PDMS matrix is actually continuously distributed. We did not detect the formation of EGaIn-Fe alloy within the LMMRE.

To study the electrical properties of the LMMRE, we investigated variables that may affect the resistivity of the material, including EGaIn droplet content and size, Fe particle content and size, curing agent/PDMS ratio, as well as curing temperature and time (see Supplementary Figs. 2–6 and Notes 1–4). Here, we briefly summarise the outcomes. For each variable, we prepared a set of samples and measured their resistivity at different strains. The optimum values of the corresponding variables were determined by considering the highest possible electrical conductivity with the lowest modulus of the LMMRE. The composite only containing PDMS and Fe particles (2–5 µm, mass fraction of 80%) is effectively an insulator, and the addition of EGaIn microdroplets into the composite significantly lowers the resistivity. We found that the optimal EGaIn microdroplet mass fraction is 20%, and the optimal droplet diameter is ~15 µm. After determining the content of EGaIn microdroplets, we examined the effect of Fe particle size (50 nm to 40 µm) and content, and chose Fe microparticles with the diameter of 2–5 µm and the overall mass fraction of 64% to provide low resistivity, low modulus, and high pressure sensitivity for the LMMRE. In addition, we surprisingly discovered that the non-conductive elastic matrix also has a great influence on the resistivity of the LMMRE. We chose PDMS (curing agent/PDMS ratio of 1:7) as the matrix to obtain the lowest resistivity. Overall, the LMMRE containing 16% PDMS (1:7), 64% Fe microparticles (2–5 µm) and 20% EGaIn microdroplets (diameter of ~15 µm) provides the optimal performance.

### Positive piezoconductive effect of the LMMRE

**Fig. 2:**
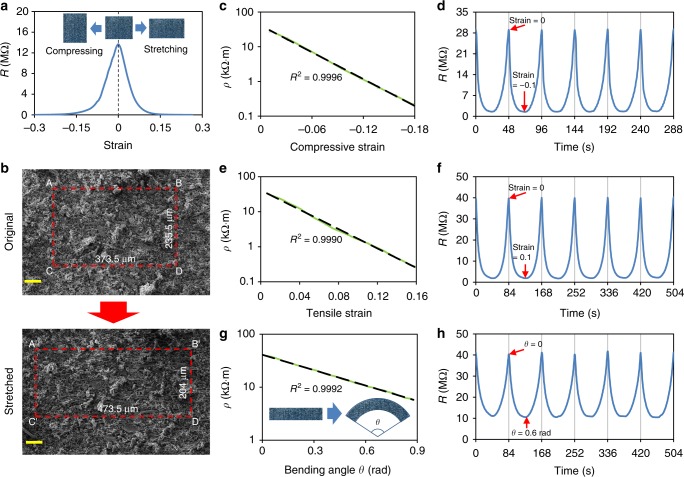
Resistivity of the liquid metal-filled magnetorheological elastomer (LMMRE) upon the application of mechanical loadings. **a** Resistance-strain curve of the LMMRE. **b** Scanning electron microscopy (SEM) images of the LMMRE under relaxed and tensile conditions (scale bars are 100 µm). Resistivity-strain curves and the resistance changes under cyclic loading under **c**, **d** compression, **e**, **f** stretching, and **g**, **h** bending

We analysed the resistance change of the optimal LMMRE under different mechanical loadings. Surprisingly, unlike conventional composites, the resistivity of this LMMRE is maximum when relaxed, and reduces sharply upon the application of either compressive or tensile strains. Figure 2a shows the resistance *R* measured for an LMMRE strip (4 × 6 × 10 mm) under compressive and tensile strains, from which we can see that its resistance lowered from 13.7 MΩ to <13 kΩ (>1000 times) with the application of a 0.25 compressive or tensile train. The resistance returned to its initial value after restoring the sample to the original dimensions.

For most conductive elastic composites, resistance increases during elongation (i.e., negative piezoconductivity) due to the increase in distance between solid metal particles^28^. Using the fluidity and high electrical conductivity of liquid metals, a few recent studies developed liquid metal-filled conductive elastomeric composites that can minimise the reduction of resistance during stretching. Some of them have electrically self-healing capabilities. When stretched, the oxide film of the liquid metal droplets in these composites will rupture. The liquid metal can flow out and form new connections with adjacent solid conductive fillers to retain the overall resistance of the composite^24–26^. In another composite material, liquid metal droplets can form a 3D Calabash Bunch conductive network structure in the elastic matrix to maintain the resistance during extension^27^. In stark contrast, the resistance of LMMRE developed in this work can be reduced by three orders of magnitude during stretching, exhibiting a positive piezoconductive effect. To clarify the principle of this unique phenomenon, we obtained SEM images of the LMMRE to observe its surface microstructure under relaxed and tensile conditions, as shown in Fig. 2b. The Poisson’s ratio υ of the material determines whether the concentration of Fe particles in the substrate changes when mechanical deformation occurs, which is crucial for the conductivity of the sample. If the concentration of Fe particles is constant, the resistivity of the material would not change substantially even without liquid metal.

To measure the Poisson’s ratio, we found four easily identifiable points in the SEM image (denoted as A, B, C, and D in Fig. 2b). As the material is stretched along the direction of AB, their positions changed (new positions are denoted as A’, B’, C’, and D’). According to the distance between those points, we calculated that the Poisson’s ratio of the sample is extremely close to 0.5 with an error of <0.1% (see Supplementary Note. 5 for details). Therefore, it can be approximated that the volume of LMMRE is conserved. Due to the constant volume, the concentration of conductive particles in the sample does not change with strain^29^. In addition, we can see from the SEM images (Fig. 1b) that these Fe particles are spherical, so we expect that the concentration of electrical contact between them is also basically unchanged during compression and stretching. This property should maintain the stability of conductive paths during deformation.

As the resistivity of the PDMS is much higher than that of iron, EGaIn, and the thin gallium oxide layer, we believe the resistivity of the LMMRE mainly depends on the thickness of the PDMS matrix along the conductive path. Due to the constant volume (the Poisson’s ratio of ~0.5), the LMMRE will always be compressed in a certain direction upon any mechanical deformation. During stretching, the EGaIn droplets deform along with the PDMS matrix, but the rigid Fe particles do not. Consequently, the Fe particle may squeeze the surrounding PDMS matrix and EGaIn droplets, leading to a sharp reduction in the thickness of the PDMS layer between them, and even enable additional Fe particles to be in direct contact with the EGaIn droplets. We believe this effect would provide more conductive paths to reduce the overall resistivity of the composite during stretching. To verify this hypothesis, we constructed a 2D model of the LMMRE based on the SEM images and the calculated Poisson’s ratio; we simulated its resistivity before and after stretching using COMSOL, as shown in Supplementary Fig. 7. Our simulation results show the reduction in the thickness of the PDMS layer between Fe particles and the EGaIn droplets upon stretching and consequently lead to a significant decrease in the overall resistivity (see Supplementary Note. 6 for detailed discussion). Owing to the elasticity of PDMS, the EGaIn microdroplets restore to their original shapes upon removing the external load.

To quantitatively analyse the resistivity variation of the material under different mechanical loads, we carried out compression, tensile, bending, and torsion tests on the LMMRE samples. Figure 2c shows the resistivity-strain curves of the sample during compression tests. With the growth of the value in strain, the resistivity *ρ* decreased exponentially. When fitted to the exponential trend line, the coefficient of determination (*R*²) of the curves in compressive and tensile states is 0.9996. Figure 2d shows an example of the resistance change of the LMMRE during the last six cycles of the cyclic compression (full test results with >100 cycles are given in Supplementary Fig. 8 and Note. 7). In this experiment, the sample was uniformly compressed by 10% and then restored to its original length at the same rate. The resistance of the LMMRE at the relax state (strain = 0) rose rapidly in the first few cycles and gradually stabilised after 15 cycles. After stabilisation, the resistance of the LMMRE dropped to only ~5% of the initial value for all cycles when applying a compressive strain of −0.1. It is also able to restore to its initial resistance after each unloading, with the error <1%. Similar to the compression test, we observed that *ρ* of the LMMRE also decreased exponentially with the growth of the tensile strain (*R*² = 0.9990), as shown in Fig. 2e. Figure 2f shows that the LMMRE also shows excellent cyclic durability for tests with tensile strain.

Figure 2g shows the plot of resistivity vs bending angle for the LMMRE. Interestingly, the curve is also well fitted with an exponential curve (*R*² = 0.9992). As the degree of bending is small, the sample can be approximated as a circular arc and the curvature is proportional to its corresponding central angle *θ*. The resistivity decreased by ~87% when *θ* is increased from 0 to 0.9 rad. The exponential reduction of the resistivity is due to the fact that the strain in any part of the sample is proportional to *θ* when bended. For the cyclic test given in Fig. 2h, the resistance of the sample can be reduced by ~75% at a *θ* of 0.6 rad and recovered to its initial value after releasing. Similarly, the resistivity of the LMMRE reduces exponentially upon the application of torsion, as detailed in Supplementary Fig. 9 and Note 8. The resistivity of the LMMRE shows an excellent exponential response and cyclic durability upon the application of any mechanical deformation, which ensures that the LMMRE is durable and can maintain a high gauge factor (~15 at the strain of ±0.025) after repeated use. In addition, we also performed the cyclic loading-unloading experiments on the LMMRE and obtained the cyclic compressive and tensile stress-strain curves, as given in Supplementary Fig. 10. We can see that LMMRE exhibits an obvious elastic hysteresis (see Supplementary Note. 9 for detailed discussion). However, the elastic hysteresis of LMMRE has no significant effect on its electrical properties. According to the resistance change curves under cyclic loading in Fig. 2, the resistance change of LMMRE during the loading and unloading process is basically symmetrical. Furthermore, the LMMRE also shows excellent cycle stability, which is beneficial to its repeated use.

### Investigating the properties of the Ni-LMMRE

**Fig. 3:**
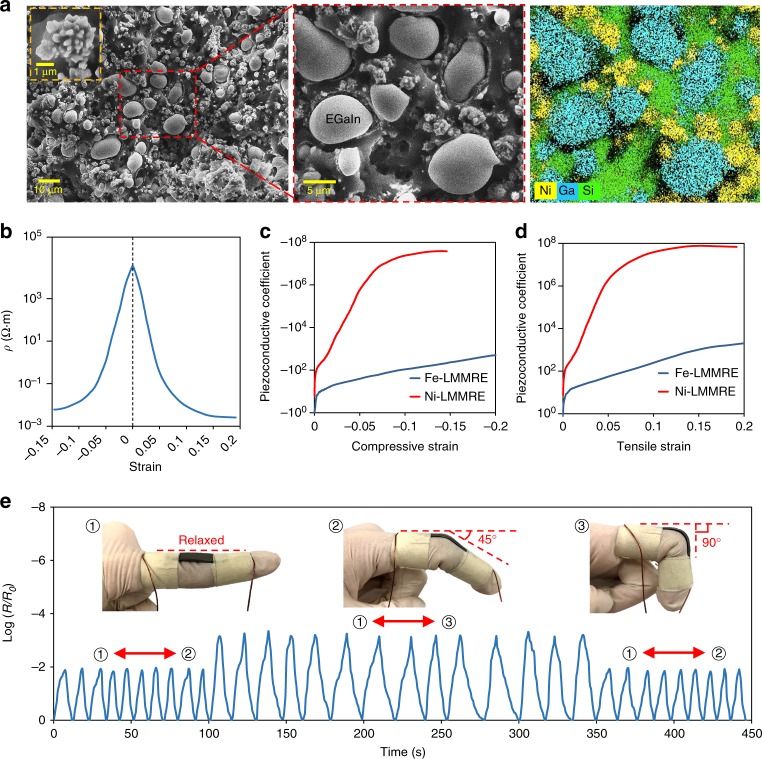
Investigating the properties of the nickel (Ni)-liquid metal-filled magnetorheological elastomer (LMMRE). **a** Scanning electron microscopy (SEM) images and the energy dispersive X-ray spectroscopy (EDS) mapping of the Ni-LMMRE (LMMRE using Ni microparticles). **b** Resistivity-strain curve of the Ni-LMMRE under compression and stretching. **c**, **d** Piezoconductive coefficient-strain curve of the iron (Fe)- and Ni-LMMRE under compression and stretching. **e** Resistance changes of the composite under cyclic bending. The strip sample was attached to the index finger

Our results show that the main factor affecting the LMMRE’s resistivity is the concentration of electrical contacts between the conductive fillers. We therefore hypothesise that replacing spherical Fe particles with irregularly shaped particles can further improve the LMMRE’s sensitivity to mechanical loadings. As the LMMRE has a Poisson’s ratio of ~0.5, the concentration of conductive particles in the incompressible material is constant. However, irregular particles have many protrusions on the surface, which provide them more opportunities to be in contact with the elongated EGaIn microdroplets, as well as with each other under stretching for forming additional conductive paths. To verify our hypothesis, we fabricated a LMMRE using irregular nickel (Ni) particles (2–5 µm) with many protrusions on its surface. The powder composed of irregular Ni microparticles is fluffier than that of the spherical microparticles. At the same mass, Ni powder is larger in volume and more difficult to be mixed in the PDMS matrix; we found that the composite cannot be formed when the Ni microparticles/PDMS mass ratio exceeds 2:1. Besides, the critical volume fraction of irregular particles in conductive composites is also lower than that of spherical particles^30^, so the mass ratio is finally determined to be 2:1 (volume ratio of Ni/PDMS is 1:4.45). Other factors such as liquid metal content, curing temperature, and curing time remained unchanged. Figure 3a shows the SEM images of the LMMRE using Ni microparticles (Ni-LMMRE). According to the EDS mapping (Fig. 3a), this composite has the same microstructure as the Fe-based LMMRE (Fe-LMMRE). The only difference is that the Fe microparticle is a smooth sphere, whereas the Ni microparticle has many granular protrusions on the surface, as shown in the inset of Fig. 3a. As a control, we fabricated a composite only containing PDMS (1 g, volume fraction of 81.6%) and Ni particles (2 g, volume fraction of 18.4%) without using EGaIn, and found that this composite is effectively an insulator (resistivity exceeds 10^6^ Ω∙m) under compressive strains smaller than –0.05.

The resistivity-strain curve for the Ni-LMMRE under compression and stretching is given in Fig. 3b. In the relaxed state, the number of electrical contacts in the composite is small and the resistivity is high (~70 kΩ∙m). However, the resistivity of the Ni-LMMRE dropped by seven orders of magnitude to <0.015 Ω∙m after applying only 0.1 compressive or tensile strain. We observed the fastest change of resistivity below the strain of 0.05. As the sample continued to be compressed or stretched, the electrical contact concentration in the composite became saturated, and the curve no longer declined exponentially. When the strain reached 0.1, the resistivity dropped to one ten-millionth of the original value. After that, the curve continued to drop exponentially at a slower rate. We compared the piezoconductive coefficient (PCC) of the Fe- and Ni-LMMRE at different strains. The PCC can be calculated as: PCC = *Δσ*/*σ*_*0*_*ε*, where *Δσ* and *σ*_*0*_ are the change in conductivity and unstrained conductivity, respectively, and *ε* is the strain of the LMMRE. As the conductivity of the LMMRE is the minimum at the relaxed state, the PCC is negative in the compressive state and positive in the tensile state, as shown in Fig. 3c, d. The PCC of Ni-LMMRE reaches its maximum of 7.88 × 10^7^ at a strain of 0.15. This PCC is five orders of magnitude higher than that of Fe-LMMRE (~300), indicating that the Ni-LMMRE is much more sensitive to mechanical deformation.

According to the tensile test results provided in Supplementary Fig. 4, the Ni-LMMRE broke when the strain reached 0.32, at which its resistivity was 100 million times less than that of the relaxed state. In addition, the high PCC allows the Ni-LMMRE to respond to extremely small mechanical deformation, which broadens its applications. For example, we applied the composite to detect the movement of human joints, as shown in Fig. 3e. We attached a Ni-LMMRE strip (1 × 5 × 30 mm) to the index finger, and bent the finger repeatedly with the maximum bending angles of 45° and 90°, respectively. Compared with the resistance in the relaxed state (*R*_0_), it reduced to only 1% at the bending angle of 45°. When the bending angle increased to 90°, the resistance dropped drastically by more than three orders of magnitude. When the bending angle was restored to 45°, the resistance change of the composite was the same as the first few cycles, indicating that its electrical property is stable under cyclic loading. Therefore, this LMMRE has the potential to be applied for developing highly sensitive flexible sensors for wearable devices.

### Response of the LMMRE to magnetic field

**Fig. 4:**
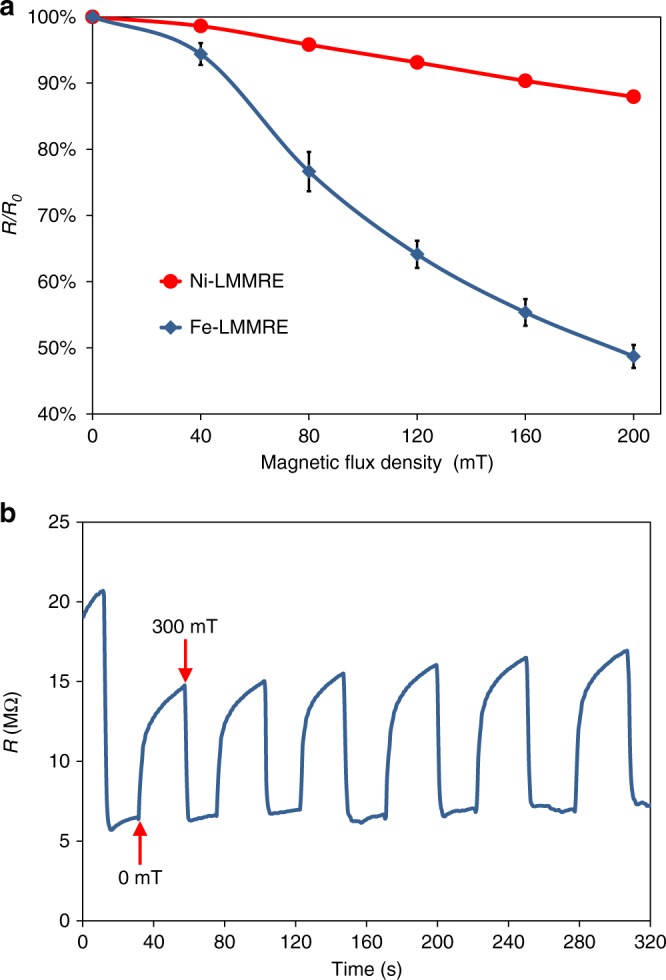
Response of the liquid metal-filled magnetorheological elastomer (LMMRE) to magnetic field. **a** The resistance changes of iron (Fe)- and nickel (Ni)-LMMRE (LMMRE using Fe and Ni microparticles) in the magnetic field. The value of the error bar is the standard deviation of the sample resistivity under five measurements. **b** The resistance change of Fe-LMMRE in periodic magnetic field

In addition to mechanical deformation, the resistivity of the LMMRE is also sensitive to magnetic field as both Fe and Ni are ferromagnetic. To study the response to magnetic field, we compressed the LMMRE by 5% and then placed it in a uniform magnetic field generated by an electromagnet. The direction of the magnetic field was perpendicular to the resistance measurement direction. Figure 4a shows the effects of the magnetic field on the resistance for both Fe- and Ni-LMMRE. For Fe-LMMRE, the resistance did not change significantly when the magnetic flux density was <40 mT. As the magnetic flux density continued to increase, the resistance change *R*/*R*_0_ dropped sharply and reached 48.7% of its original value at 200 mT. However, the magnetic field had little effect on the Ni-LMMRE because the magnetic permeability of Ni is much lower than that of Fe (Fig. 4a). We also conducted cycling tests for the Fe-LMMRE, as shown in Fig. 4b. The resistance first dropped rapidly to approximately 6 MΩ at a magnetic field of 300 mT. After removing the magnetic field, the resistance slowly increased to 15 MΩ. This process is repeatable and the resistance change *R*/*R*_0_ at 300 mT was maintained at ~40%.

We believe the decrease in resistance upon subjecting to a magnetic field can be attributed to two main reasons. First, Fe particles in the substrate tend to align along the direction of the magnetic field, which reduces the spacing of the particles and improves the electrical conductivity. Second, the sample can be deformed within a magnetic field, which is caused by magnetostriction^31^, and the increase in strain results in a reduction in resistance. A previously reported magnetorheological elastomer (MRE) can also achieve a high sensitivity to magnetic field by forming chain structures of particles using an external magnetic field before solidifying the composite^9^. In contrast, the LMMRE in our study does not require complicated fabrication steps and can maintain the isotropic properties. Using the field-responsive property of the LMMRE, we developed sensor circuits that incorporate the LMMRE to adjust both DC and AC signals in response to magnetic field, as detailed in Supplementary Figs. 11, 12 and Notes 10, 11. As such, the LMMRE possesses the potential to be used as sensors for measuring the magnetic flux density and pressures.

### Applications of the LMMRE in intelligent heating devices

Along with the high sensitivity to strain and magnetic field, the Fe- and Ni-LMMRE developed in this work also have good thermal conductivity. We compared the thermal diffusivity *α* of the Fe-/Ni-LMMRE with a mixture of PDMS and EGaIn (PDMS-EGaIn), a mixture of PDMS and Fe microparticles (Fe-MRE), and a mixture of PDMS and Ni microparticles (Ni-MRE). The details of the experimental setup and discussion are given in Supplementary Figs. 13–15, Table 2 and Note. 12. We set the volume ratio of PDMS matrix and metal fillers of the five composites to 1:0.45. We found that the Ni-LMMRE has the highest *α* due to the irregular shape of Ni particles with a large thermal conductivity (90.7 W m^−1^ K^−1^). Compared with spherical Fe particles, the irregular Ni particles with granular protrusions on the surface have more opportunities to contact with each other, and thus can significantly improve the thermal conductivity of the composite, providing advantages for its application in flexible heating devices.

**Fig. 5:**
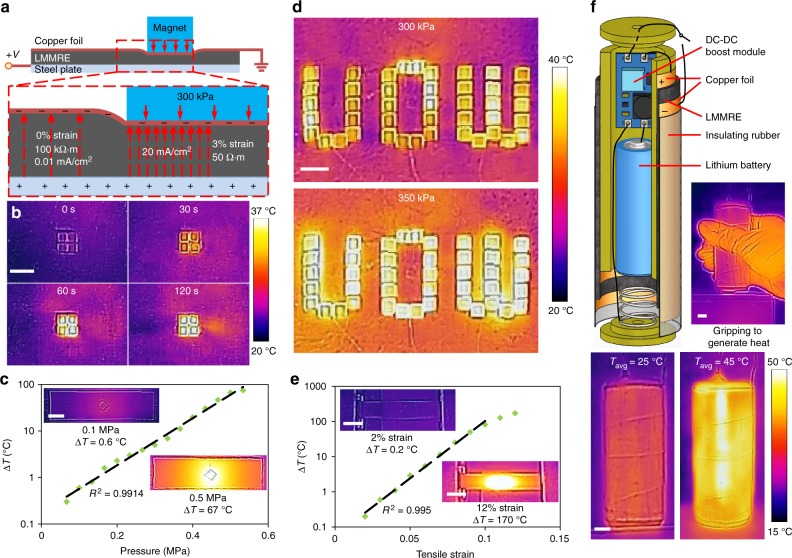
Applications of the liquid metal-filled magnetorheological elastomer (LMMRE) in heating devices. **a** Operating principle of the pressure-sensitive heating device. **b** Temperature change on the film at different times after applying magnets. **c** Temperature change *vs* pressure plot of the heating device. **d** Localised heating effect of the device. **e** Temperature change *vs* tensile strain plot. **f** Exploded schematics and thermal images of the hand-held heating column. Scale bars are 1 cm

To harness the unique properties of LMMRE, we developed an intelligent pressure-sensitive heating device. The operating principle of this heating device is illustrated in Fig. 5a. The core component of the device is a 1 mm thick film made of Ni-LMMRE. The lower surface of the film is attached to a thin steel plate and the upper surface is covered with a layer of copper foil. With the application of a 10 V voltage to the steel plate and grounding the copper foil, electrical current with greater current density will be generated through regions with lower resistance, inducing Joule heating effect. In doing so, we placed four cubic magnets (N50, 4 × 4 × 4 mm) on the surface of the film and applied a 10 V potential between the steel plate and the copper foil to demonstrate this process. The area without magnet has a high resistivity of 100 kΩ·m, and the current density at this area (0.01 mA cm^−2^) is insufficient to generate significant heat. In contrast, the magnet locally compressed the sample, causing a drop in resistivity to only ~50 Ω·m. The current density in the compressed zone increased to 20 mA cm^−2^, which induced a heating power of 0.2 W cm^−2^. Figure 5b shows that the temperature of the magnet rose dramatically from 23 to 33 °C within 1 min (heating rate of ~0.17 K s^−1^), and remained stable at 35 °C after 2 min. The cyclic compression experiment of the device is shown in the Supplementary Fig. 16 and Note. 13. As the *α* of the film is much lower than that of the steel plate, the excess heat would be dissipated through the steel plate after reaching the equilibrium temperature, ensuring that the un-pressed film around the magnet would not be heated simultaneously.

In addition to the heating rate, we also investigated the relationship between the final temperature and the pressure applied to the film by placing a square polymethyl methacrylate (PMMA) sheet (side length of 10 mm) on the film and loaded different weights on it to yield different pressures. Figure 5c shows that the equilibrium temperature increased exponentially (*R*² > 0.99) with respect to the applied pressure, reaching a high temperature of 90.1 °C at a pressure of 0.5 MPa (corresponding to a current density of 0.3 A cm^−2^). To further demonstrate the localised heating effect on this LMMRE, we spelled out the word “UOW” on the film using 43 cubic magnets. In 2 min, they were heated from 23 to ~35 °C, as shown in Fig. 5d. After cooling to room temperature, we placed the film on a large steel block and repeated the experiment. This induces a larger magnetic force and therefore, the surface pressure of the film increased to 350 kPa. As such, the equilibrium temperature increased to 40 °C in this experiment (Fig. 5d). This simple device can heat the object on the pressed area and automatically adjust the temperature according to the pressure. In addition, the device has no complicated circuit structure or uses any pressure sensor, which reduces the risk of damage and makes it easy to repair. After repeated tests, there was no damage or crack observed at the edge of the pressed area on the film. Most importantly, this device can produce different temperatures simultaneously in multiple areas without affecting each other.

Due to the positive piezoconductive characteristic of the LMMRE, this heating film can also produce heat during stretching based on the Joule heating effect when applying a voltage. In doing so, we fixed two ends of a Ni-LMMRE film (5 × 1 × 30 mm) to a pair of electrodes and powered it using a 30 V DC source. The film was stretched by 0.01 strain each time and maintained for 2 min to measure the corresponding equilibrium temperature, as recorded in Fig. 5e. The film temperature started to rise when the strain was larger than 0.02, and the temperature increased exponentially with respect to the applied strain (*R*^2^ = 0.995), reaching a high temperature of 190 °C at the tensile strain of 0.12. We observed that at high tensile strain, the induced high temperature started to affect the resistance and slow down the growth rate of temperature.

As no wires or sensors are needed within the composite, this heating film can be cut into any shape and work on a curved surface. Based on this advantage, we developed a hand-held heating column based on this Ni-LMMRE film. Figure 5f shows the exploded view of the heating column. The heating film was sandwiched between two copper foils and wrapped around a plastic cylinder. The cylinder was 3D printed and has a diameter of 3.5 cm and a height of 10 cm. The device was powered by a 3.7 V lithium battery and the voltage was boosted to 28 V by a DC-DC boost module. The positive and negative output electrodes were respectively connected to the copper foil on both sides of the film. The device was covered with a layer of insulating rubber to maintain heat and protect the device. When using the heating column, the user only needs to turn on the switch and gently apply the appropriate gripping force to the device (Fig. 5f). As we can see from the thermal images that the initial average temperature of the film was 25 °C; and its temperature rose to 45 °C after holding it in hand for 1 min. This device can work for about 3 h with a lithium battery with the capacity of 1800 mAh, it can also be used multiple times (see Supplementary Figs 16, 17 and Notes 13, 14 for details). The desired temperature of the device can be easily controlled by the gripping strength based on the user’s requirement.

## Discussion

We report a LMMRE composite that possesses numerous unique properties. Compared with conventional composites, the unique property is that this LMMRE exhibits a positive piezoconductive effect, whose resistivity is maximum in the relaxed state, and drops drastically and exponentially with the application of any mechanical deformations, including stretching, compression, bending, and twisting. We thoroughly explored the effects of particle, elastomer, liquid metal content, liquid metal droplet size, and curing process on the physical properties of the LMMRE. For LMMRE using irregular Ni metal particle fillers, its resistivity can be reduced to ten-millionth of its initial value with the application of a strain <0.2, reaching a high PCC of 7.88 × 10^7^. Almost all of the previously reported composites do not have this unique property to the best of our knowledge. The LMMRE can therefore have both a high PCC and a low electrical resistance during elongation. In addition, the LMMRE is also magnetic field responsive that can significantly reduce its resistivity within magnetic field. Apart from investigating the effects of the solid microparticle fillers, modifying the surface properties of liquid metal, such as coating it with a layer of semiconductive or conductive nanoparticles^32–34^, may also affect the overall electrical properties of the composite.

Apart from electrical properties, we discovered that the LMMRE with irregular fillers also has superior thermal conductivity in comparison with conventional composites with spherical fillers. Harnessing the unique properties of the LMMRE, we demonstrated several proof-of-concept applications including fabricating a pressure-sensitive smart heating device using the LMMRE film. This device can adjust the temperature by applying pressure via gripping, which not only identifies and heats the area that is in contact with user’s hand, but also automatically stops working when pressure is removed. This feature may enable flexible or wearable heating devices like intelligent heating pads and heated insoles. Owing to the excellent mechanical, electrical, and thermal properties, the LMMRE has the potential to be further developed for forming future innovative functional and flexible devices with superior performance.

## Methods

### Materials and preparation of the elastic composites

EGaIn liquid metal and hydroxy iron powder were purchased from Sigma-Aldrich, Australia. SYLGARD® 184 Silicone Elastomer Curing Agent and SYLGARD® 184 Silicone Elastomer Base were purchased from Dow Corning, American. The copper, Ni, zinc, silver, molybdenum and cobalt powder were purchased from NAIYATE Alloy welding material Ltd., China.

When preparing the sample, we first weighed the raw materials and placed them in a plastic test tube with a diameter of 15 mm in the order of PDMS-Fe powder-EGaIn, then stirred the mixture using a high-speed electric stirrer (rotating speed ranging from 400 to 2000 rpm) equipped with a plastic stick (diameter of 4 mm) for 5 min. After that, we vacuumed the mixture for 15 min to remove air bubbles and poured it into a mould made of PMMA board. The mould was placed in the oven (70 °C) for 6 h to obtain the LMMRE.

### Experimental equipment and tools

A Screw Driven Linear Guide is used to form mechanical deformation of the LMMRE sample to measure its electrical characteristics. The two ends of the LMMRE sample were pasted with copper electrodes and fixed on the Linear Guide. In the compression and stretching test, the block sample (6 × 10 × 10 mm) and strip sample (4 × 6 × 10 mm) were uniformly compressed or stretched with a speed of 0.4 mm min^−1^. In the bending test, the size of the strip sample was 3 × 6 × 40 mm. A VICI Digital Multimeter (VC8145) is used to measure electrical data such as the resistance of the composite and the current of the heating device. The digital multimeter has a resistance range of 100 MΩ, so it can measure a resistivity of one million Ω∙m for the block sample (6 × 10 × 10 mm). The MTS Landmark 370.02 hydraulic load frame is used to measure the stress-strain curve and Young’s modulus of the composites. SEM images were obtained using a JEOL JSM-6490LA SEM. A Cat S60 FLIR infrared thermal camera was used to obtain the thermal images and videos. COMSOL Multiphysics 5.1 software package (Burlington, MA, USA) was used to simulate the distribution of Fe microparticles and EGaIn microdroplet upon stretching, as well as calculate the change of resistivity of the LMMRE.

## Supplementary information

Supplementary Information

Peer Review File

## Data Availability

The authors declare that the main data supporting the findings of this study are available within the article and its Supplementary Information files. Extra data are available from the corresponding author upon request.
