# Supplementary material for: Liquid metal-filled magnetorheological elastomer with positive piezoconductivity
Source: Nat Commun. 2019 Mar 21;10:1300. doi: 10.1038/s41467-019-09325-4 (PMC6428896; doi:10.1038/s41467-019-09325-4)
Supplement: Supplementary file 1 — Supplementary Information [file 41467_2019_9325_MOESM1_ESM.pdf]

Supplementary Information

Liquid Metal-filled Magnetorheological Elastomer with Positive Piezoconductivity

Yun et al.

Supplementary Figures

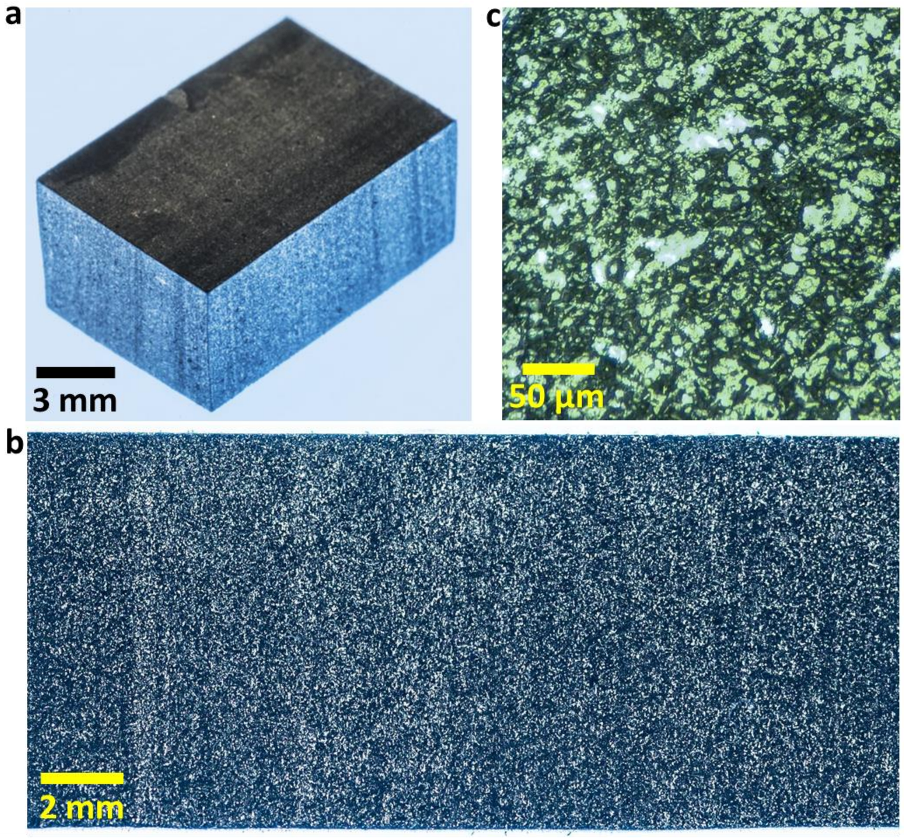

Supplementary Figure 1. Optical image of the LMMRE. Optical images of the LMMRE obtained using **a-b** a single lens reflex (SLR) camera and **c** an optical microscope. The white spots in the image are EGaIn droplets. The overall colour of the LMMRE is silver grey.

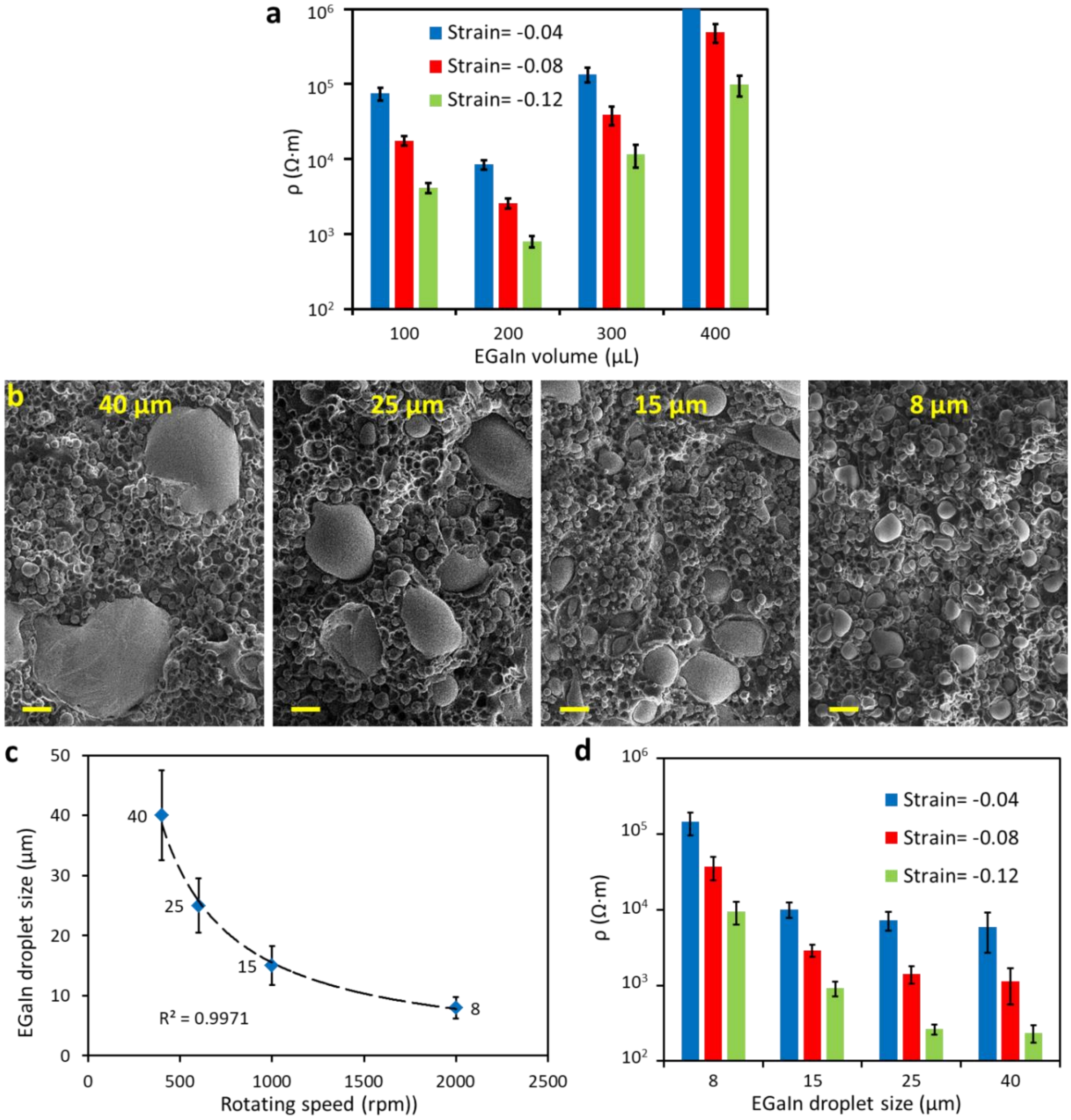

Supplementary Figure 2. Influence of the content and droplet size of EGaIn on the electrical properties of the LMMRE. a Resistivity of the LMMRE with different EGaIn contents. The value of the error bar is the standard deviation of the sample resistivity under 5 measurements.**b** SEM images of LMMRE samples with different EGaIn droplet diameters. Scale bars are 10 μm. **c** Average diameter of EGaIn droplets in LMMRE *vs* stirring speed. The value of the error bar is the standard deviation of the diameters of all EGaIn droplets in the SEM image. **d** Resistivity of the LMMRE with different EGaIn droplet diameters. The value of the error bar is the standard deviation of the sample resistivity under 5 measurements.

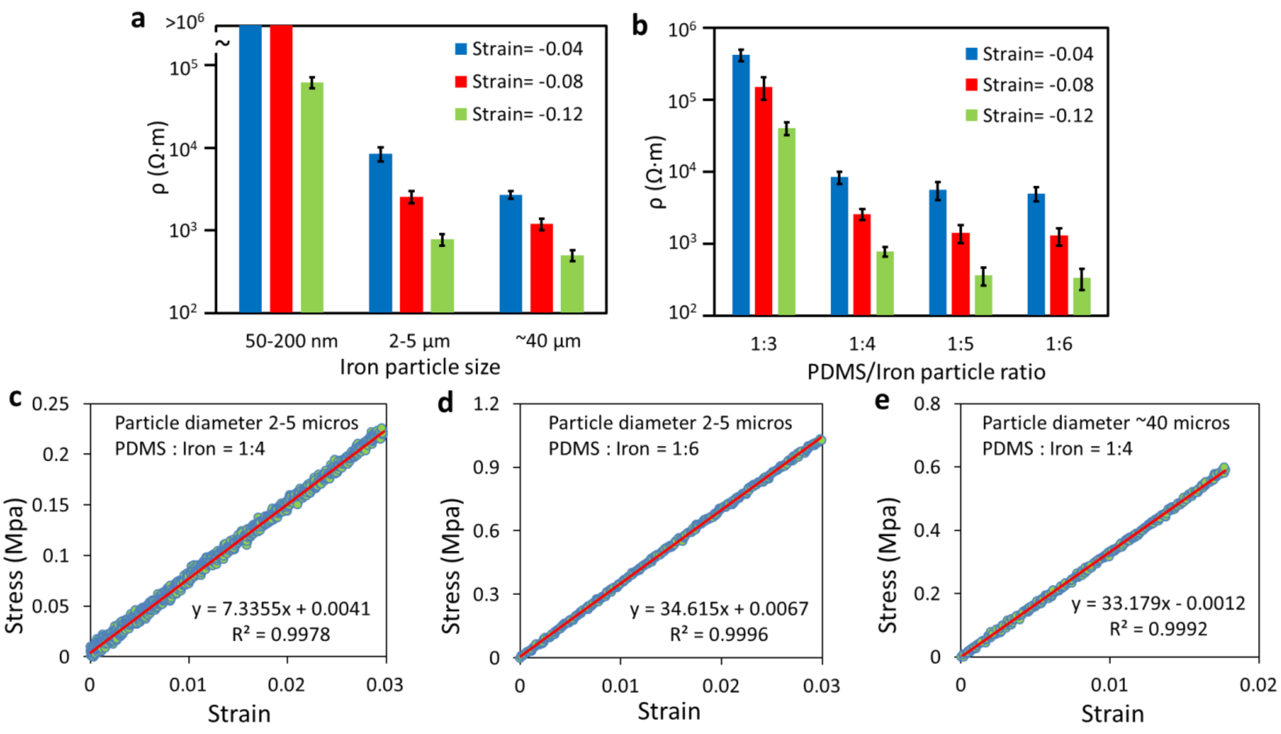

Supplementary Figure 3. Influence of the content and size of Fe particles on the electrical properties of the LMMRE. Resistivity of the LMMRE prepared using **a** Fe particles with different sizes, and **b** different Fe particle contents. The value of the error bar is the standard deviation of the sample resistivity under 5 measurements. Stress-strain curves of the LMMRE with **c** Fe particle diameter of 2-5 μm and PDMS/Fe particle mass ratio of 1:4. **d** Fe particle diameter of 2-5 μm and PDMS/Fe particle mass ratio of 1:6. **e** Fe particle diameter of ~40 μm and PDMS/Fe particle mass ratio of 1:4. We performed a least square fit on the three stress-strain curves. The slope of the fitted line was the Young's modulus of the corresponding material. The Young's modulus of the three materials are 7.336, 34.615 and 3.179 MPa, respectively.

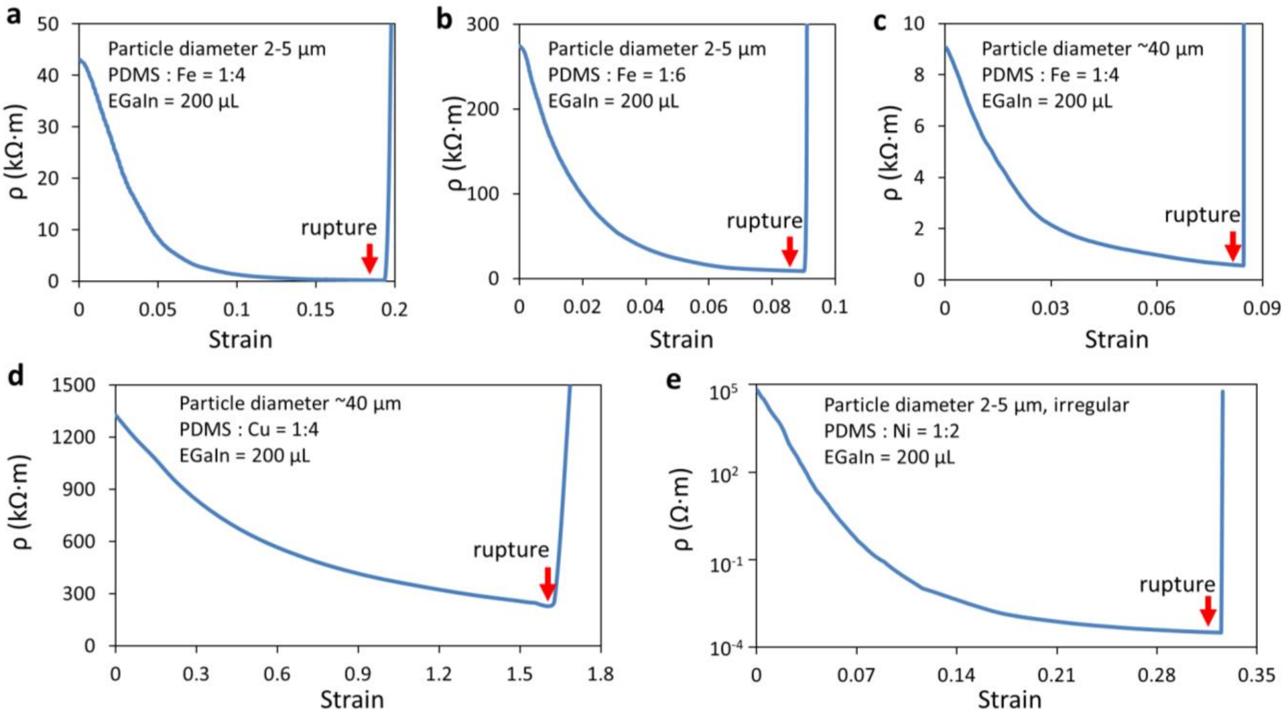

Supplementary Figure 4. Tensile limit of LMMRE with different fillers. Resistivity-strain curves of the LMMRE produced with **a-b** Fe particles with the diameter of 2-5 µm; the sample with the PDMS/Fe ratio of 1:4 broke after applying a nearly 20% of strain, whereas for the sample with the ratio of 1:6 broke only after 9% of strain. Resistivity-strain curves of the composites produced with **c** Fe particles with the diameter of 40 µm, **d** Cu particles, and **e** irregularly shaped Ni particles.

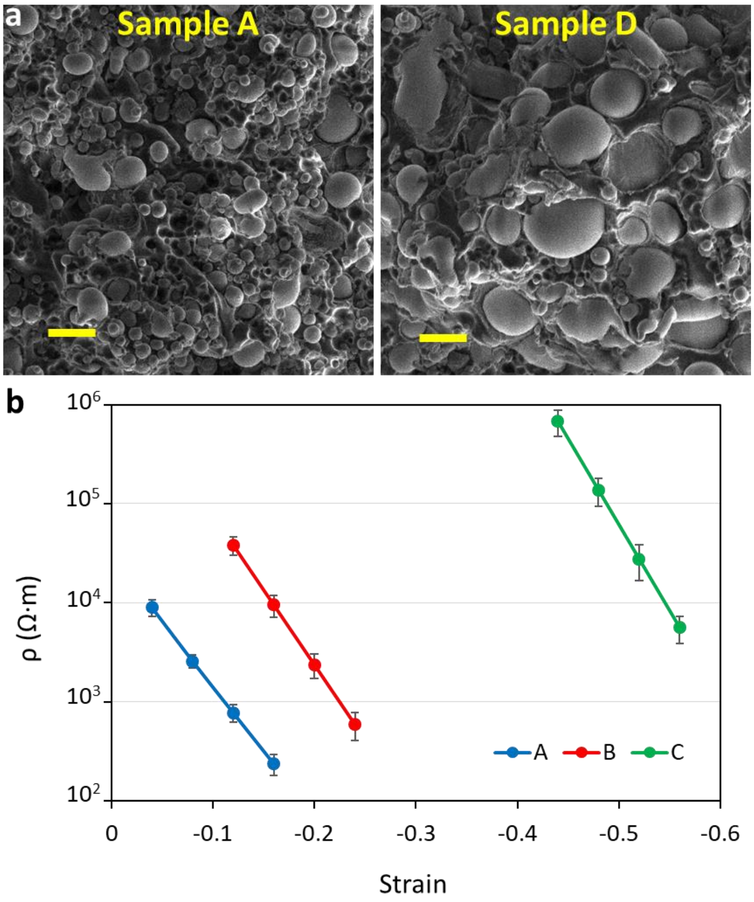

Supplementary Figure 5. The effect of the relative content of iron particle and EGaIn on the electrical properties of LMMRE. a SEM images of Samples A and D. Scale cars are 10 µm. **b** Resistivity of the Fe-LMMRE prepared with different iron particle and EGaIn contents *vs* compressive strain. The value of the error bar is the standard deviation of the sample resistivity under 5 measurements.

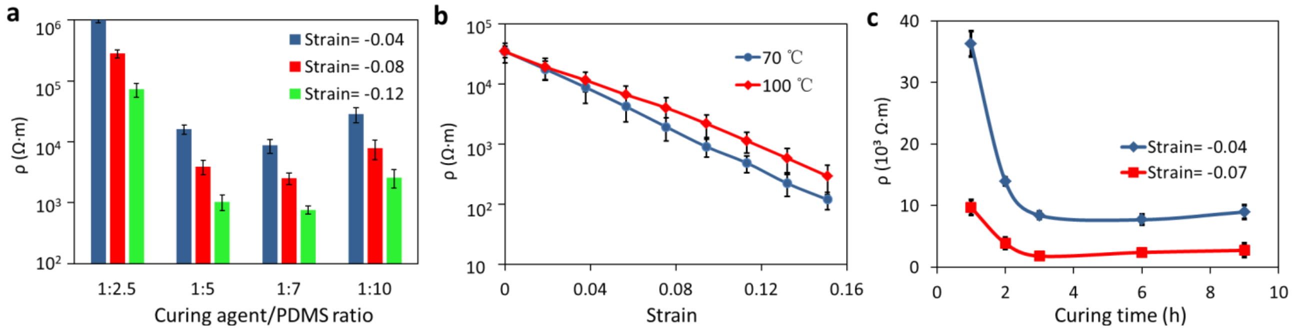

Supplementary Figure 6. Additional variables that affect the resistivity of the LMMRE. Resistivity of the Fe-LMMRE prepared using **a** PDMS with different curing agent contents, **b** different curing temperatures, and **c** different curing time. The value of the error bar is the standard deviation of the sample resistivity under 5 measurements.

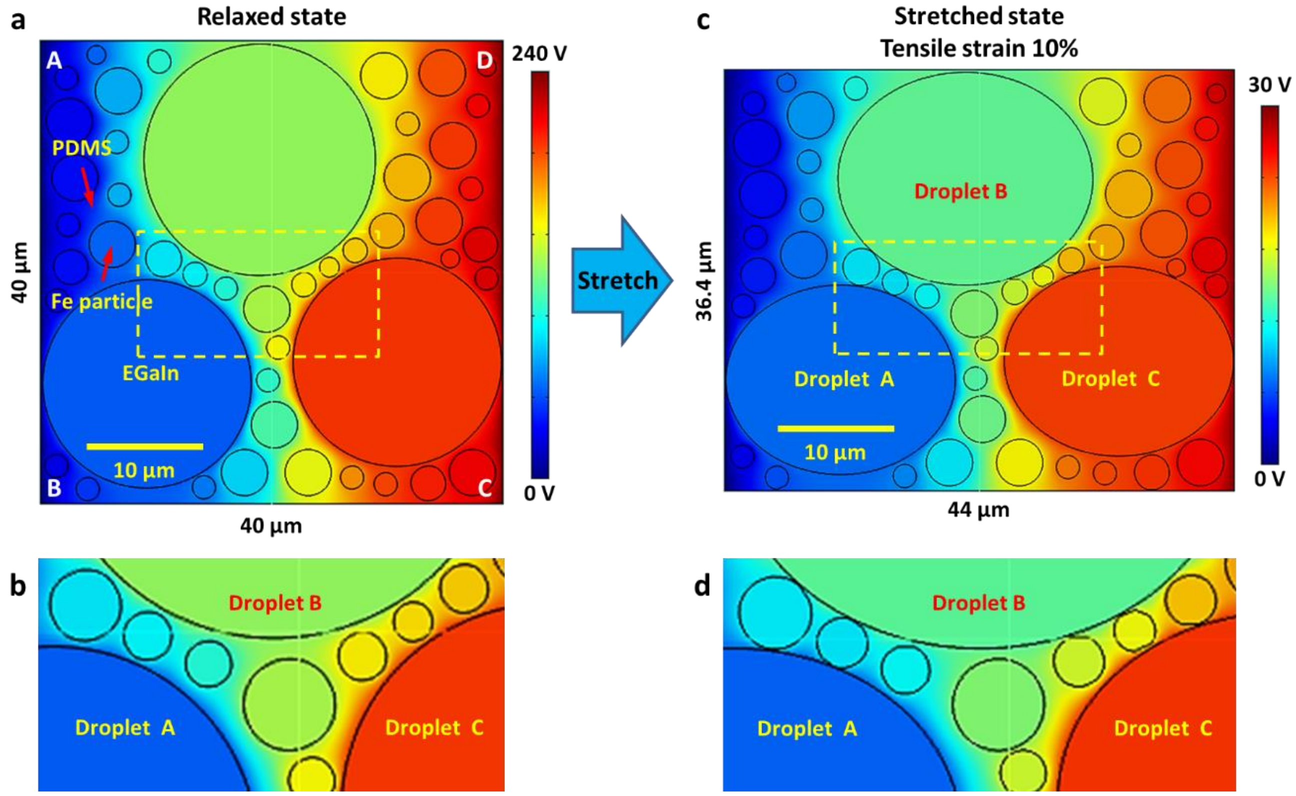

Supplementary Figure 7. Numerical simulation of the sheet resistance of the LMMRE before and after stretching. Simulation results of the electrical potential distribution of the LMMRE when passing through a constant electrical current density of 125 A/m^2 **a** before and **c** after stretching. **b** and **d** show the enlarged view of the yellow-dashed area in **a** and **c**, respectively.

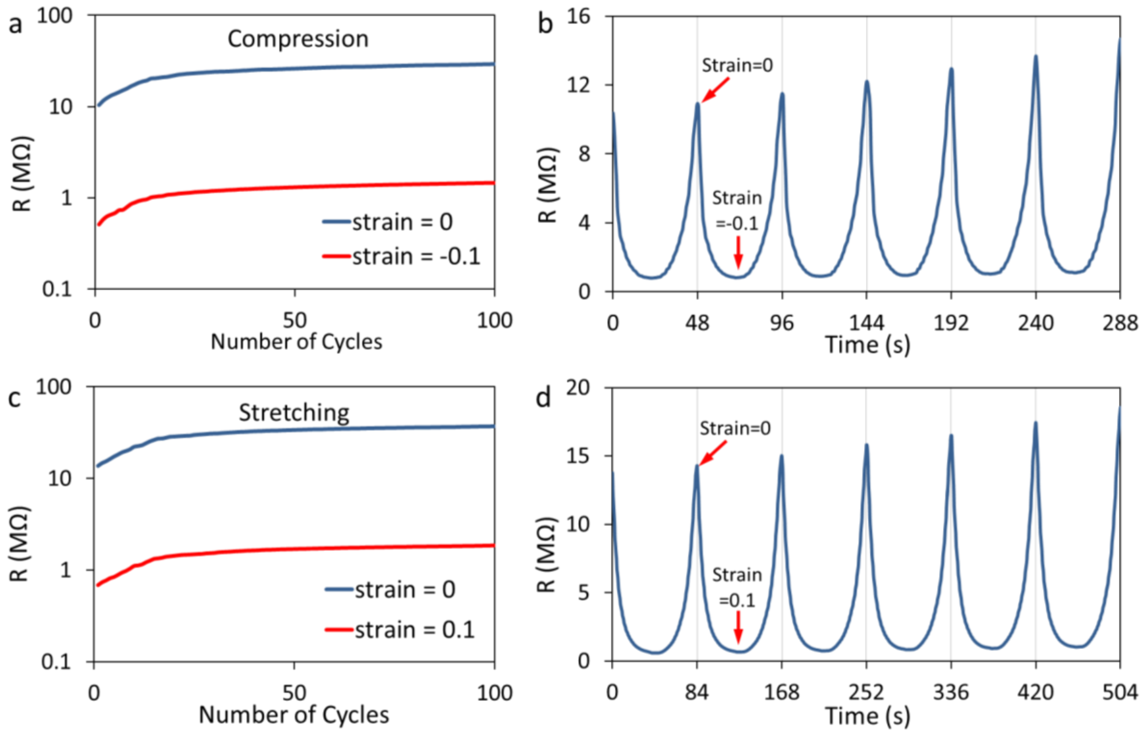

Supplementary Figure 8. Change of resistance for the LMMRE under cyclic loading. Resistance changes of LMMRE under **a** cyclic compression load and **c** cyclic tensile load. Resistance changes of the LMMRE during the first six cycles of **b** cyclic compression load and **d** cyclic tensile load.

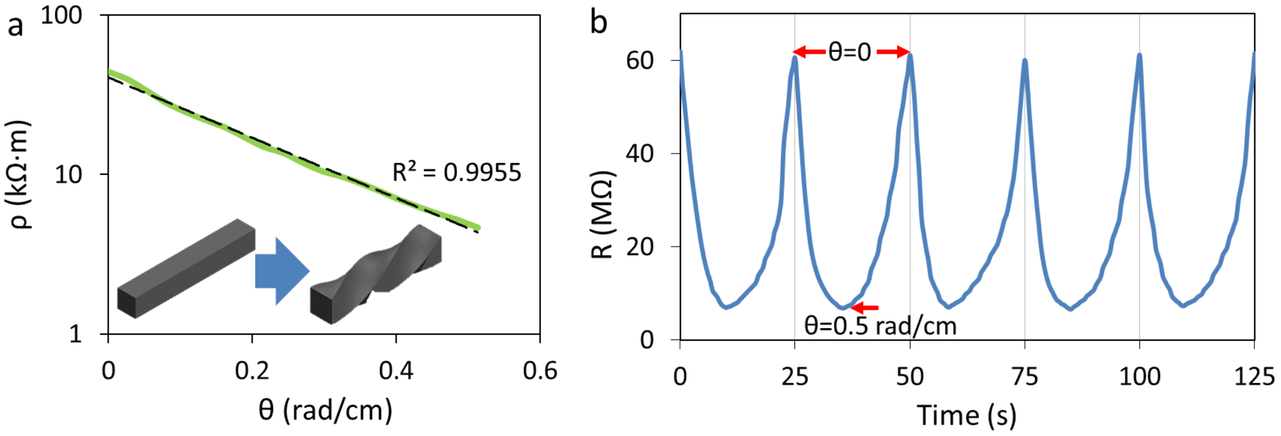

Supplementary Figure 9. Change of resistivity for the LMMRE in torsion test. a Resistivity-twist angle curve of a LMMRE strip. **b** Resistance change of sample in a cyclic torsion test.

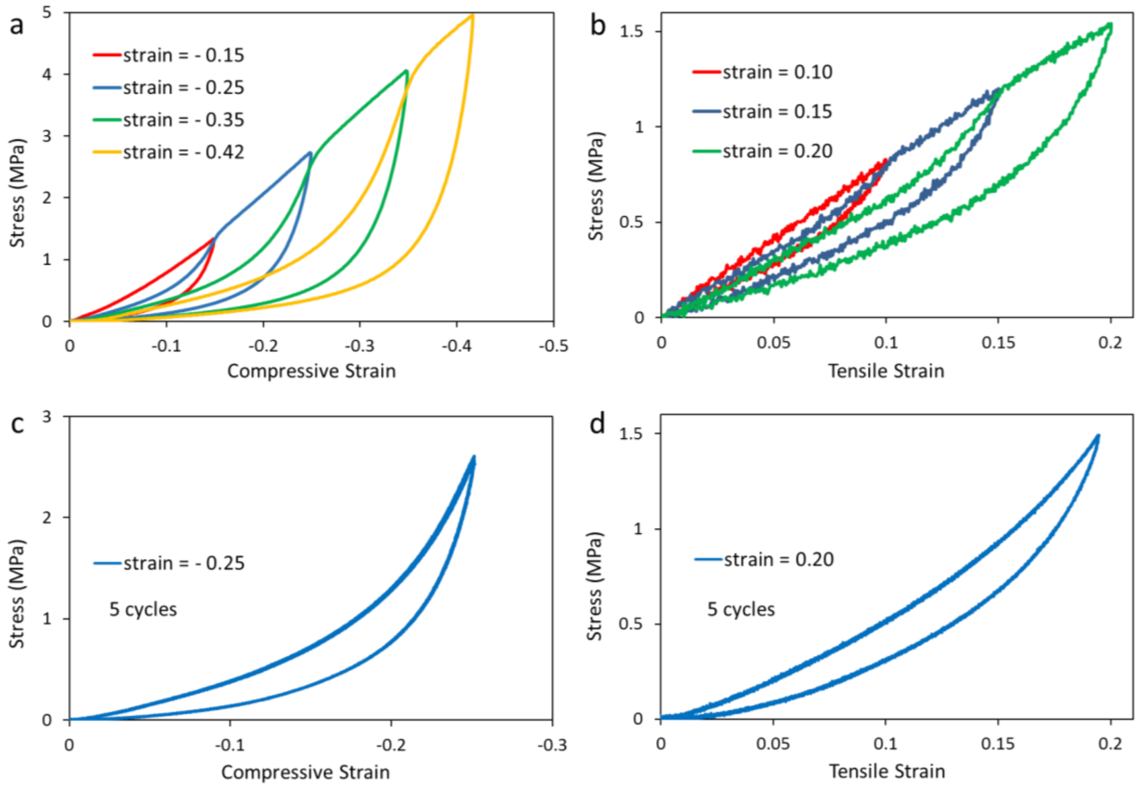

Supplementary Figure 10. Cyclic loading-unloading tests of the LMMRE. Cyclic **a** compressive and **b** tensile loading–unloading plots of LMMRE with increasing strains. Cyclic **c** compressive and **d** tensile loading–unloading plots of LMMRE to the same maximum strain.

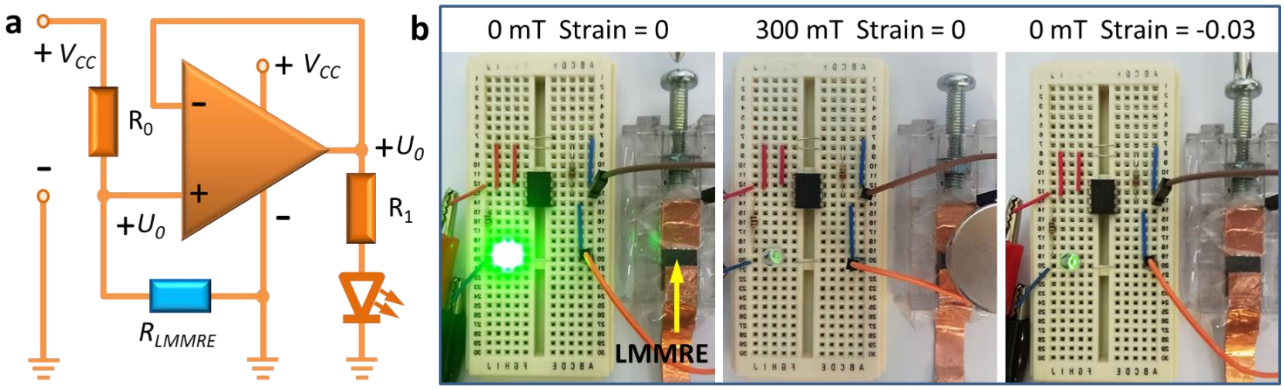

Supplementary Figure 11. LMMRE-based circuit for detecting magnetic field and strain. a Diagram and **b** actual experiment for the circuit that utilizes the Fe-LMMRE to detect the change of magnetic field and strain.

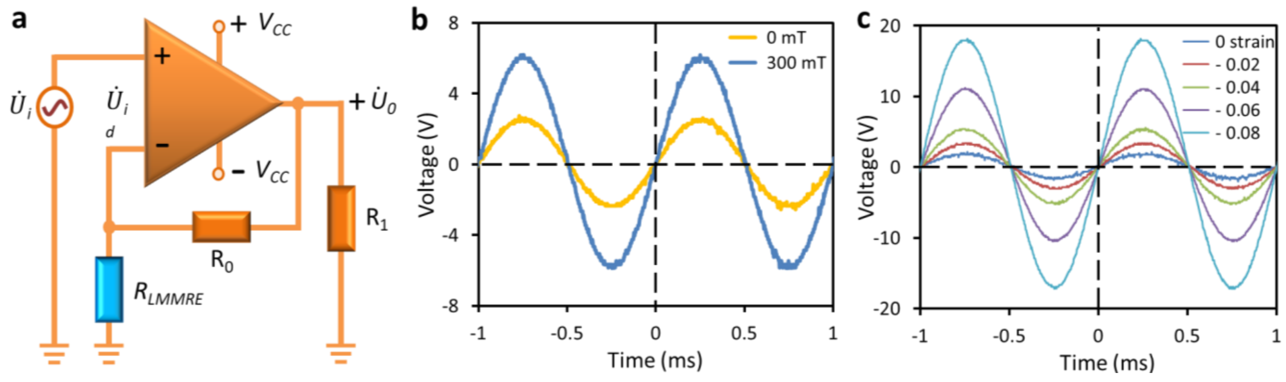

Supplementary Figure 12. LMMRE-based AC signal amplification circuit. a Diagram and **b** actual output signals for the circuit that utilizes the Fe-LMMRE to control the amplification of the input AC signal.

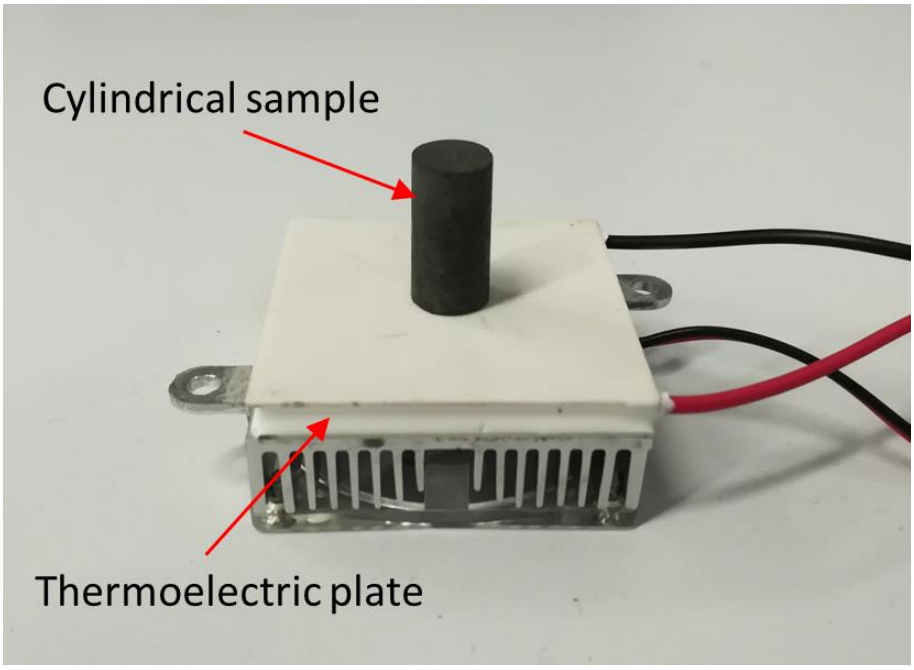

Supplementary Figure 13. Experimental setup of the thermal conduction experiment. The bottom surface of the samples was fixed on a thermoelectric plate using thermal conductive adhesive.

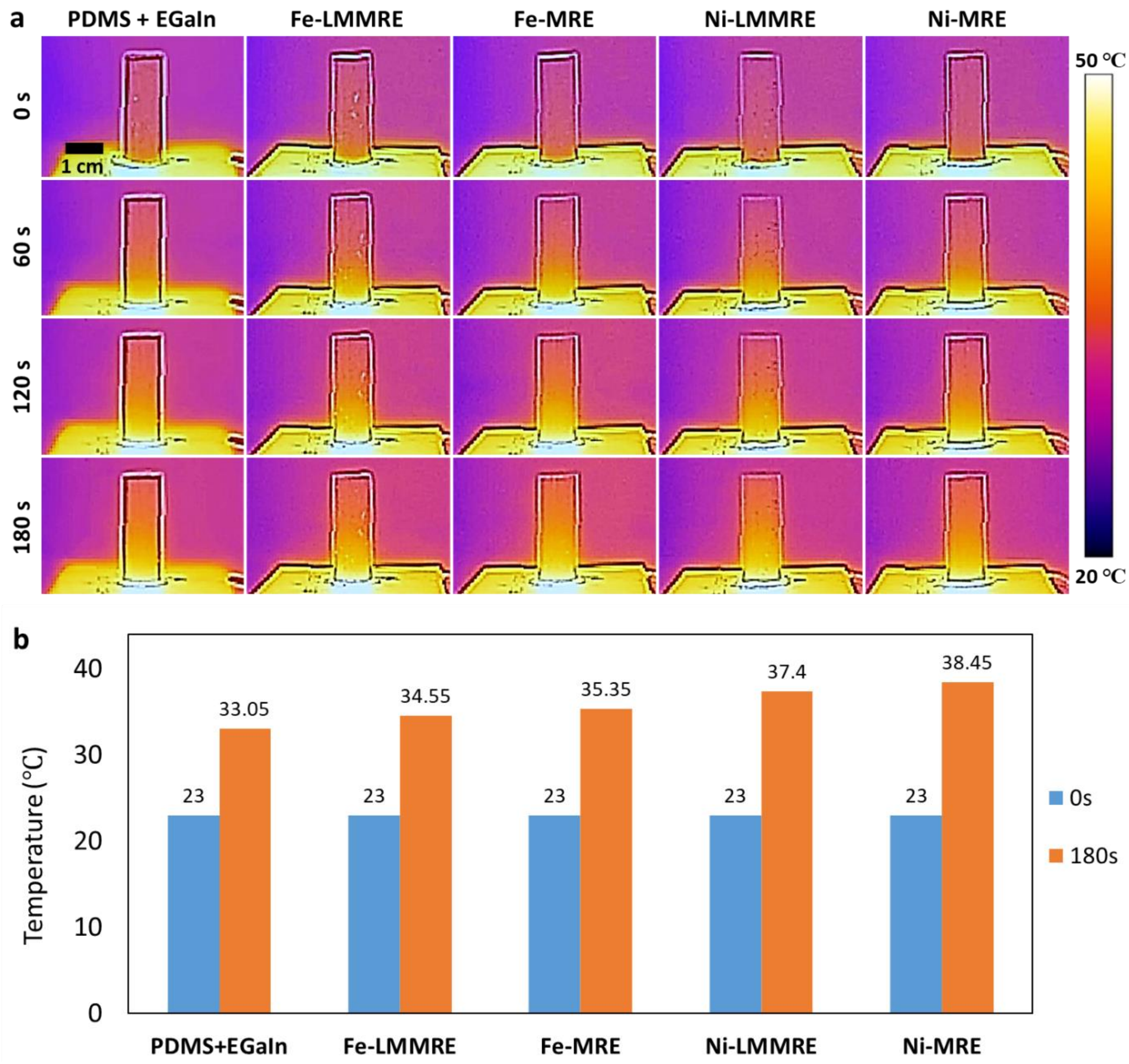

Supplementary Figure 14. Thermal conduction experiments of five composites. a Thermal images of the cylindrical PDMS-EGaIn composite, Fe-/Ni-MRE, and Fe-/Ni-LMMRE on a thermoelectric plate. **b** Temperature change at the centre of the five samples before and after the experiment. In the thermal conduction experiment, the initial temperature of the five cylindrical samples was 23 °C. The temperature at the centre of these samples at 0 and 180 seconds were recorded as shown in the Figure.

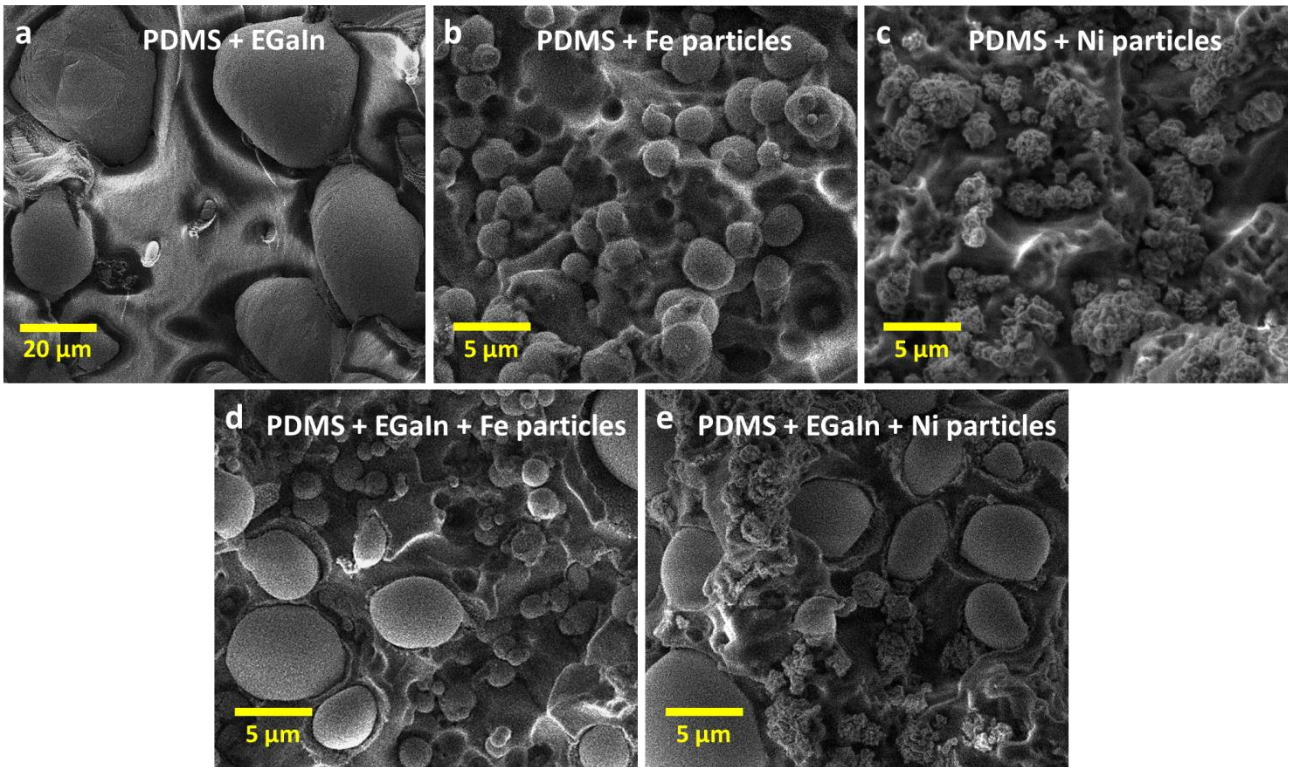

Supplementary Figure 15. SEM images of the PDMS-EGaIn, Fe-MRE, Ni-MRE, Fe-LMMRE, and Ni-LMMRE.

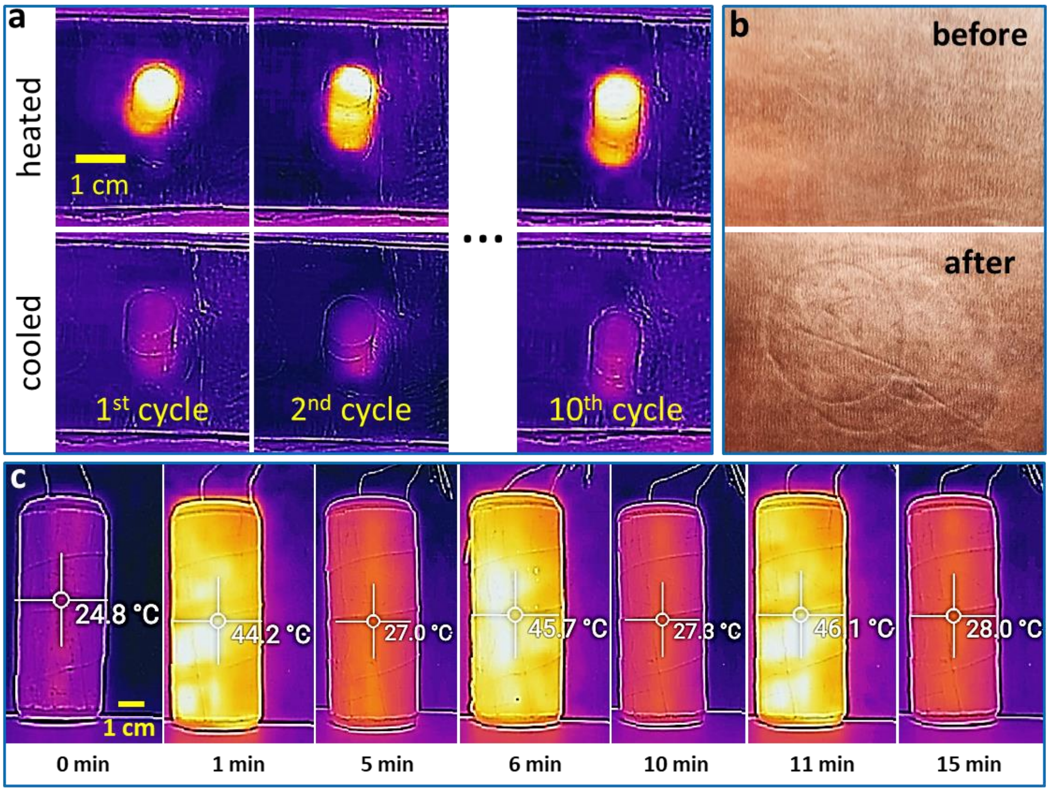

Supplementary Figure 16. Cyclic compression experiments of the heating devices. Thermal images of the cycle compression experiments of **a** the pressure-sensitive heating device and **c** the hand-held heating column. **b** The photographs of the copper foil on the surface of the heated film before and after the cyclic compression experiment.

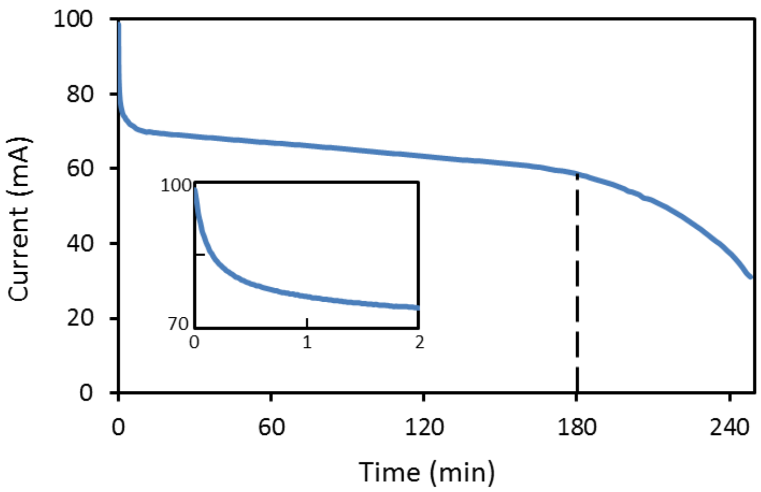

Supplementary Figure 17. Working duration of the hand-held heating column. Current *vs* time curve of the hand-held heating column powered by a lithium battery (1800 mAh). The inset shows the current-time curve for the first two minutes.

Supplementary Tables

Supplementary Table 1. Qualities and mass fractions of materials contained in samples A-D.

Sample	PDMS	Fe particles	EGaIn
Sample A	1 g (16%)	4.0 g (64%)	1.25 g (20%)
Sample B	1 g (16%)	3.5 g (56%)	1.75 g (28%)
Sample C	1 g (16%)	3.0 g (48%)	2.25 g (36%)
Sample D	1 g (16%)	2.5 g (40%)	2.75 g (44%)

Supplementary Table 2. Density, thermal conductivity and volumetric heat capacity of five composites.

	PDMS-EGaIn	Fe-LMMRE	Fe-MRE	Ni-LMMRE	Ni-MRE
ρ (g/cm^3)	2.608	2.885	3.105	2.965	3.269
c_p (J/(g·K))	0.623	0.645	0.660	0.633	0.639
ρc_p (J/(cm^3·K))	1.625	1.861	2.049	1.877	2.089

Supplementary Notes

Supplementary Note 1: Influence of the content and droplet size of EGaIn on the electrical properties of the LMMRE

We firstly examined the effect of EGaIn on the electrical properties of the LMMRE. We fabricated a composite only containing PDMS (1 g, volume fraction of 66.2%) and Fe particles (2-5 μm, 4 g, volume fraction of 33.8%), and found that this material is effectively an insulator (resistivity exceeds 10^6 Ω·m) under the compressive strains of –0.04, –0.08 and –0.12. The negative strain represents a state of compression. The resistivity of the composite reduces significantly after mixing with EGaIn during the production process, as shown in Supplementary Figure 2a. It can be seen from the SEM image (Fig. 1b of the main manuscript) that there are a large number of EGaIn droplets distributed in foam-like cavities that are not connected to each other. This geometry prevents the direct conduction between EGaIn droplets and adjacent Fe particles. Upon subjecting the composite to external forces, the cavities enclosing the EGaIn are squeezed, resulting in a decrease in the distance between EGaIn droplets and adjacent Fe particles. Owing to the high electrical conductivity of EGaIn, the EGaIn microdroplets can act as a bridge for connecting the Fe particles, and consequently reduce the overall resistivity. Since we only applied small compressive strains (less than 20%), we did not observe the formation of destructive cracks within the LMMRE. We discovered that the resistivity of the LMMRE sharply decreases as the EGaIn content increases from 0 to 200 μL. However, when the volume of EGaIn exceeds 200 μL, further increasing its volume somehow significantly increases the resistivity of LMMRE, for reasons we do not yet understand. For composite containing 400 μL EGaIn, the resistivity can exceed 10^6 Ω·m at the strain of −0.04 (cf. Supplementary Figure 2a). We also found that LMMRE containing more EGaIn makes the resistance measurement relatively unstable (indicated by the larger error bars). Therefore, using 200 μL of EGaIn not only greatly reduces the resistivity but also improves the stability of measurement.

In addition to the content, the size of the EGaIn droplets also has a great influence on the electrical properties of the composite. We prepared four LMMRE samples using 1 g PDMS, 4 g iron powder and 1.25 g EGaIn (PDMS/Fe particle/EGaIn volume ratio of 1:0.51:0.2). To analyse the influence of EGaIn droplet size on the electrical properties of LMMRE, we mixed the raw materials under different rotating speeds of the electric stirrer to control the EGaIn droplet size within the LMMRE. Due to the large surface tension of EGaIn, a large shear force is required during the stirring process to break it into microdroplets. The shearing force depends on many factors such as the stirring speed, the size of the stirring head and the viscosity of the liquid. Due to the same ratio of raw materials, the viscosity of the mixed liquids of the four samples is basically the same. We used a cylindrical plastic stick (diameter of 4 mm) as the stirrer and stirred the mixture for 5 minutes at different rotating speeds (400, 600, 1000, 2000 rpm, respectively). We measured the size of the EGaIn droplets in the four LMMRE samples (Supplementary Figure 2b) and obtained their relationship to the rotating speed (Supplementary Figure 2c). Obviously, the diameter of the EGaIn droplet is inversely proportional to the rotating speed (R^2 = 0.9971). This is because the droplet size is inversely proportional to the shear force, which is proportional to the linear velocity at the edge of the stirring head. Since the diameter of the stirring head is constant, the line speed is proportional to the rotating speed.

Supplementary Figure 2d shows the resistivity of these LMMRE samples. As we can see, LMMRE with smaller EGaIn droplets has higher resistivity. In LMMRE, the EGaIn droplets can connect adjacent Fe particles and form conductive pathways to improve the conductivity of the composite. This phenomenon is especially significant when the EGaIn droplet is much larger than the Fe particles (diameter 2-5 µm). If the diameter of the EGaIn droplets is too small (< 10 µm), the amount of Fe particles that it can connect will decrease drastically, resulting in a rapid rise in the resistivity of the composite. However, large droplets create large cavities in the composite that are easily broken under mechanical deformation and cause EGaIn to seep out. This impairs the mechanical

strength and the resistivity stability of the composite (indicated by the larger error bars in Supplementary Figure 2d). Therefore, we choose the stirring speed of 1000 rpm in the subsequent sample fabrication to achieve both the low resistivity and the high stability of measurement. In this case, the EGaIn droplet diameter is about 15 μm.

Supplementary Note 2: Influence of the content and size of Fe particles on the electrical properties of the LMMRE.

We investigated the effect of Fe particles on the electrical and mechanical properties of the LMMRE. We prepared the LMMRE samples using 1 g PDMS (1:7), 200 μL EGaIn, and 4 g Fe particles with different sizes. Supplementary Figure 3a presents the effect of Fe particle size on material resistivity. For composite using Fe nanoparticles with a diameter of 50-200 nm, we obtained a very large resistivity exceeding 10^6 Ω·m when applying −0.04 and −0.08 compressive strains. This result occurs because the mechanical strength and the number of connections required for creating the conductive path within the LMMRE increases when using smaller particles[1]. The material resistivity decreases sharply when increasing the size of the Fe particles. Nonetheless, we found using large particles compromises the mechanical properties of the LMMRE. We obtained stress-strain curves and resistivity-tensile strain curves for LMMRE prepared using Fe particles with diameters of 2-5 μm and ~40 μm, as shown in Supplementary Figure 3. The Young's modulus of the latter is 33.18 MPa, which is 4.5 times higher than that of the former (the measured Young's modulus of the 1:7 PDMS is ~2.6 MPa). Such a high stiffness reduces its pressure sensitivity and also makes the material more prone to tear. Upon subjecting the material to an external force, Fe particles with a larger size can cause uneven deformation and severe stress concentration, reducing the strength of the material. We finally chose Fe microparticles with the diameter of 2-5 μm to provide both low resistivity and high pressure sensitivity for the LMMRE.

After determining the diameter of the Fe particles, we next investigated the effect of the particle concentration on the resistivity of the LMMRE. We prepared the samples using 1 g PDMS (1:7), 200 μL EGaIn, and Fe particles (2-5 μm) with different concentrations. When the mass ratio of PDMS/Fe particle is 1:3, the LMMRE is almost electrically insulated at rest, as shown in Supplementary Figure 3b. The resistivity drops dramatically by nearly two orders of magnitude when we increase the mass ratio to 1:4. However, the resistivity of the LMMRE does not significantly decrease even after increasing the ratio to 1:6. This can be attributed to the fact that the Fe particle concentration may already exceed its critical volume fraction for conduction in composite. In addition, the Fe particle content also affects the stiffness of the composite. We obtained the stress-strain curves and calculated the Young's modulus of LMMRE samples with different Fe particle contents, as detailed in Supplementary Figure 3. According to the stress-strain curves, the Young's modulus of samples with PDMS/Fe particle mass ratios of 1:4 and 1:6 are 7.34 and 34.62 MPa, respectively. When subjected to stress, stiffer materials have less deformation and resistance change, and therefore are less desirable for fabricating pressure-sensitive devices. Additionally, we found that in comparison to low-Fe particle content materials, high-Fe particle content materials are more brittle and cannot withstand large tensile strains (see Supplementary Figure 4 for details). Based on the above-mentioned considerations, we chose a PDMS/Fe particles mass ratio of 1:4 for the LMMRE.

Supplementary Note 3: Resistivity of the LMMRE prepared using different Fe particle and EGaIn contents

To further analyse the influence of the relative content of Fe particle and EGaIn on the electrical properties of the composite, we prepared four LMMRE samples and compared their electrical properties. These four samples contain 1 g PDMS and 5.25 g metal filler (including iron powder and EGaIn). The qualities and mass fractions of raw materials contained in these samples are given in Supplementary Table 1. The SEM images (Supplementary Figure 5a) show the large difference in

EGaIn content in Samples A and D. We measured the resistivity-strain curves of the four samples, as shown in Supplementary Figure 5b. As the content of Fe particles decreased and the content of EGaIn increased, the resistivity of the samples rose sharply. Sample D was always electrically insulated at any strain and would break when the compressive strain exceeds 60%. We can see that LMMRE containing 64% Fe particles and 20% EGaIn (i.e. 1 g PDMS, 4 g Fe particles and 1.25 g EGaIn) has the lowest resistivity, which is consistent with our previous conclusions.

Supplementary Note 4: Additional variables that affect the resistivity of the LMMRE

Although PDMS is non-conductive, we surprisingly discovered that it also has a great influence on the resistivity of the LMMRE. We prepared the LMMRE samples using 1 g PDMS, 4 g Fe particles (2-5 μm), and 200 μL EGaIn. We found that if the curing agent/PDMS ratio is too high (1:2.5), the viscosity of the mixed liquid will become too low to significantly shear EGaIn into small droplets during stirring. This will consequently increase the overall resistivity and stiffness of the final composite. In addition, the low viscosity could also result in sedimentation of Fe particles and EGaIn microdroplets during the curing process. On the other hand, higher ratios of curing agent to PDMS may be desirable as it can lead to a faster curing time to minimize precipitation. To solve this contradiction, we eventually chose the curing agent/PDMS ratio of 1:7 and found that the resistivity is the lowest at such a ratio, as shown in Supplementary Figure 6.

In addition to the materials used for preparing the LMMRE, we found that the curing temperature and time also affect the electrical properties of the material. It takes several days for the mixed liquid to solidify at room temperature, so it is necessary to heat it to speed up the process. To determine the appropriate heating temperature, we cured two samples at 70 and 100 °C for 6 h, respectively, and obtained their resistivity-strain curves (Supplementary Figure 6b). Although their resistivity is similar in the relaxed state, the higher curing temperature may slightly weaken its sensitivity to

compression. So we set the heating temperature at 70 °C. At this temperature, the mixed liquid takes around 1 h to solidify. However, the conductivity of the sample is extremely poor after 1 h curing. As the heating time increases to three hours, we observed a significant drop of the resistivity and it remains relatively steady even we prolonged the curing time to 9 h (Supplementary Figure 6c).

In addition to PDMS (Sylgard-184), we also tested other elastomers such as Ecoflex as the encasing material for the LMMRE. We found that Ecoflex is easy to deform when subjected to stress because of its low Young's modulus (~0.1 MPa)[2]. Upon the application of an external force, the interior of the material may greatly deform and eventually tear to form interconnected gaps[2]. The liquid metal that originally connected the metal particles can flow along these gaps and even seep out of the surface, causing the disconnection of metal particles and the rapid increase in resistance. Besides, at the same strain, the resistivity of the Ecoflex-based LMMRE is ~10,000 times higher than that of the PDMS-based composite. After comparison, we believe Sylgard-184 is more suitable than Ecoflex for forming functional LMMRE.

Supplementary Note 5: The calculation of the Poisson's ratio of the LMMRE

A, B, C and D are four easily identifiable points in the SEM image of the LMMRE sample (cf. Fig. 2b in the main manuscript). As the material is stretched along the direction of AB, the positions of the four vertices of the rectangle also changed (new positions are denoted as A', B', C', and D'). The distance of AB increased from 373.5 to 473.5 μm, while the length of BD was laterally compressed from 235.5 to 204 μm upon stretching. The Poisson of the material can be calculated as:

$$\nu = -\frac{\varepsilon_{trans}}{\varepsilon_{axial}} = -\frac{\frac{B'D'}{BD} - 1}{\frac{A'B'}{AB} - 1} \approx 0.50 \qquad (1)$$

where ε_{trans} is the transverse strain (perpendicular to the tensile direction), and ε_{axial} is the axial strain (in the tensile direction).

Supplementary Note 6: Simulation of the resistance of LMMRE before and after stretching

To simulate the change of resistivity before and after stretching, we drew a 40 × 40 μm LMMRE 2D plane model based on the obtained SEM image and import it into COMSOL for simulation. The Poisson's ratio of the PDMS matrix was set at 0.5, the Fe particles were treated as rigid circles, and the volume of the liquid metal droplets remained constant during stretching. After stretching, the amount of longitudinal elongation of the PDMS and EGaIn droplets in the model equals their transversal compression ratio. EGaIn droplets change from circular to oval shape. Since the Young's modulus of Fe particles is much larger than that of PDMS matrix, Fe particles are spherical before and after stretching, but its centroid position changes along with the PDMS matrix. In the simulation, the AB side of the composite is set to ground and the current density that passes through the CD side is set constant at 125 A/m2, whereas no current flows through the BC and AD sides. According to the simulation results, the sheet resistance of the LMMRE before and after stretching was 23.99 GΩ/sq and 2.451 GΩ/sq, respectively. The reason for the sharp drop in sheet resistance may be found in the voltage distribution of LMMRE before and after stretching, as given in Supplementary Figure 7. Since the resistivity of the PDMS matrix is much higher than that of iron, EGaIn and its thin surface oxide layer, the resistivity of the composite depends on the thickness of the PDMS matrix on the conductive path. As shown in Supplementary Figure 7b, in the relaxed state, there is a thicker PDMS barrier between EGaIn droplet A, B and the Fe particles between them. The potential difference between droplets A and B is ~100 V. When the sample is stretched in the longitudinal direction, it is compressed in the lateral direction, and the distance between the droplets A and B reduces (Supplementary Figure 7d). Since the Fe particles maintain a spherical shape and constant diameter during the stretching process, they will squeeze the EGaIn droplets A and B. This may result in a sharp decrease in the thickness of the PDMS layer between the droplets A, B and the Fe particles (see Supplementary Figures 7b and 7d). At this time, the potential difference between the

droplets A and B is only ~10 V, and the overall sheet resistance of the composite also reduces greatly. In 3D composites, this phenomenon of reduced resistance will be more obvious.

In principle, no matter what kind of mechanical deformation the LMMRE composite is subjected to, it will always maintain a constant volume (the Poisson's ratio of ~0.5), and be compressed in a certain direction. Although the PDMS thickness between the metal fillers increases slightly along the stretching direction, the distance between the EGaIn droplets and the metal fillers in the transversal and direction is drastically reduced because of the undeformed Fe particles. We believe this reduces the thickness of the PDMS layer along the conductive path and the overall resistivity of the composite. In our future work, we will conduct further experimental investigation using high-resolution 3D scanning instrument such as X-ray computed tomography facility (e.g. nano-CT) to fully verify the change of the microstructures of the LMMRE during deformation.

Supplementary Note 7: Change of resistance for the LMMRE under cyclic loading

In this cyclic experiment, the sample was uniformly compressed/stretched by 10% and restored to its original length >100 times. In the compression test, the sample had an initial resistance (strain = 0) of 10.4 MΩ. Then the resistance began to rise rapidly (Supplementary Figure 8b) and gradually stabilized after 15 cycles. At the end of the experiment, the resistances at the relaxed state and under a −0.1 compressive strain were maintained at 30 and 1.5 MΩ, respectively. A similar phenomenon was observed in the tensile test. The sample resistivity in the relaxed state was stabilized after 15 cycles. The increase in resistivity during cyclic test could be attributed to the redistribution of EGaIn microdroplets within the LMMRE.

Supplementary Note 8: Change of resistivity for the LMMRE in torsion test

In the torsion test, one end of the strip sample was fixed and the other end was evenly twisted around the central axis of the sample. The relationship between the resistivity of the sample and the twisting angle per centimetre is shown in Supplementary Figure 9a. The curve fit well to the exponential trend line curve ($R^2 = 0.9955$). Under torsion, the strain at any point in the bar is proportional to the twist angle. Therefore, in this test the material resistivity still decreased exponentially with the increase of strain.

In cyclic torsion test, the sample resistance was about 60 MΩ at the relaxed state. In each cycle, as the twisting angle increased to 0.5 rad/cm, the resistivity decreased exponentially to 7 MΩ. Then it returned to 60 MΩ after the sample was restored to its original state.

Supplementary Note 9: Cyclic loading-unloading tests of the LMMRE

In the cyclic loading-unloading experiments, we applied a cyclic load to the LMMRE sample using the MTS Landmark 370.02 hydraulic load frame to measure its stress-strain behaviour. In Supplementary Figures 10a and 10b, the maximum load for each cycle is gradually increased, while the maximum load for each cycle in Supplementary Figures 10c and 10d remained constant. We can see that the LMMRE exhibits obvious elastic hysteresis. Besides, the Young's modulus of the LMMRE in the relaxed state decreases as the maximum load increases. Under the same maximum load, the stress-strain curves of LMMRE in 5 cycles are completely coincident (Supplementary Figures 10c and 10d), indicating that the LMMRE has an excellent cycle stability.

Supplementary Note 10: LMMRE-based circuit for detecting magnetic field and strain

Using the LMMRE, we developed a simple negative feedback circuit that can detect pressure and magnetic field. Supplementary Figure 11a is its circuit diagram. R_0 and R_1 are fixed resistors with resistance of 1 MΩ and 10 Ω, respectively. R_{LMMRE} is a Fe-LMMRE sample with an initial resistance of 100 kΩ. DC power supply voltage V_{cc} was 5 V. We used an operational amplifier (op-amp) to generate a negative feedback, and the output voltage U_0 is proportional to the resistance of R_{LMMRE}. Before applying an external magnetic field, R_{LMMRE} has the maximum resistance and the LED lamp was bright; the LED lamp immediately dimmed once the magnetic field was detected, as show in Supplementary Figure 11b. The brightness of the LED gradually decreased as we increase the intensity of the magnetic field, and the LED was eventually extinguished when the magnetic flux density reached 400 mT. In addition to the magnetic field, the brightness of the LED lamp can also be controlled with pressure. The LED gradually dimmed to extinguish as the compressive strain increased from 0 to 0.03.

Supplementary Note 11: LMMRE-based AC signal amplification circuit

Supplementary Figure 12a is the circuit diagram of the LMMRE-based AC signal amplification circuit controlled by the magnetic field and strain. The resistance of R_0 and R_1 were 2.3 and 1 MΩ, respectively, and V_{cc} was 20 V. The input signal U_i is a sinusoidal signal with a frequency of 1 kHz and amplitude of 100 mV. The signal amplification factor can be calculated as $f = (R_0+R_{LMMRE})/R_{LMMRE}$. The value of f increased from 24 to ~60 with the application of a magnetic field with the intensity of 300 mT, and the amplitude of the output signal increased to ~6 V. Compared to magnetic fields, the output signal was more sensitive to strains, as detailed in Supplementary Figure 12c. A high amplification factor of 180 was obtained with the application of a 0.08 compressive strain to the LMMRE.

Supplementary Note 12: Comparison of thermal diffusivities of five composites

We compared the thermal diffusivity α of the Fe-/Ni-LMMRE (PDMS/Fe(Ni) particle/EGaIn volume ratio of 1:0.25:0.2) with a mixture of PDMS and EGaIn (PDMS-EGaIn, PDMS/EGaIn volume ratio of 1:0.45), a mixture of PDMS and Fe microparticles (Fe-MRE, PDMS/Fe particle volume ratio of 1:0.45), and a mixture of PDMS and Ni microparticles (Ni-MRE, PDMS/Ni particle volume ratio of 1:0.45). According to the recent study by Tutika et al., the volume fraction of metal fillers in composites has a great effect on the thermal conductivity of the material[3]. To reflect the difference in thermal conductivity of different types of composites, we set the volume ratio of PDMS matrix and metal fillers of the five composites to 1:0.45. The experimental setup is shown in Supplementary Figure 13. The initial temperature of the samples was 23 °C and the temperature of the plate was maintained at 50 °C. All of these samples were cylindrical and equal in diameter (10 mm) and height (23 mm). We monitored their temperature change using an infrared thermal camera within the first three minutes, as shown in Fig. 5a (see Supplementary Figure 14a for details). We compare the temperature changes at the centre of the five samples before and after the experiment (cf. Supplementary Figure 14b), in which we can see that the Ni-LMMRE and Ni-MRE have the fastest temperature rise (~15 °C temperature change in 3 min) in comparison to other materials. We know that α can be determined as: $\alpha = k/\rho c_p$, where k is thermal conductivity, ρ is density, and c_p is specific heat capacity. Together, ρc_p is the volumetric heat capacity which describes the ability of a unit volume of a material to store internal energy when undergoing a given temperature change (without undergoing a phase transition). To calculate the volumetric heat capacity, we need to know the density and specific heat capacity of each material. Density can be obtained by weighing the mass of a known volume of material, as given in Supplementary Table 2. The specific heat capacity of a composite is equal to the mass-weighted average of the specific heat capacities of the individual components, which can be calculated as below:

$$c = \frac{\sum_{i=1}^{n} m_i c_i}{\sum_{i=1}^{n} m_i} \quad (2)$$

The specific heat capacities of PDMS, Nickel, Iron, Gallium and Indium at room temperature are 1.46, 0.44, 0.44, 0.37 and 0.23 J/g·K, respectively. According to this formula and the mass ratio of each component in the five materials, we can calculate their specific heat capacity and volumetric heat capacity as given in Supplementary Table 2.

Although metal particles slightly increase the material density, the difference in the volumetric heat capacity of the five samples is insignificant. Therefore, their thermal diffusivity is mainly determined by their thermal conductivity *k*.

The *k* of EGaIn is only 26.6 W/m·K, this makes the temperature of PDMS-EGaIn sample changed slowly. In addition, due to the lack of metal particle filler, the viscosity of the mixed liquid is low and thus does not shear the liquid metal significantly during stirring. As a result, the size of the EGaIn droplets is large (30-50 μm in diameter), as shown in the SEM image given in Supplementary Figure 15a. The PDMS between adjacent EGaIn droplets could be as thick as tens of microns, so the thermal conductivity of the PDMS-EGaIn composite is the smallest among the five materials.

The SEM images (Supplementary Figures 15b and 15c) show that the distribution of Fe and Ni particles in Fe- and Ni-MRE sample is quite dense, which in turn reduced the limitation of the PDMS on the heat transfer and improved the thermal conductivity. Supplementary Figures 15d and 15e show that Fe- and Ni-LMMRE have the similar microstructure. Since the difference of *k* between Fe (80.2 W/m·K) and Ni (90.7 W/m·K) is very small, we believe that the large difference in thermal conductivity between Fe-composites and Ni-composites is due to the irregular geometry of Ni particles. At the same concentration, the irregular Ni particles with granular protrusions on the surface have more opportunities to contact with each other than the spherical Fe particles, which in turn can significantly improve the thermal conductivity of the composite. Although the thermal

conductivity of Ni-MRE is slightly higher, Ni-LMMRE has much lower Young's modulus and resistivity, which provides advantages for its application in flexible heating devices.

Supplementary Note 13: Cyclic compression experiments of the heating devices

We conducted cyclic compression tests for the heating devices given in Figs. 5b and 5g. For the pressure-sensitive heating device in given Fig. 5b, we first placed a plastic cylinder (1 cm diameter) on the film and applied a pressure of about 400 kPa. The cylinder is heated to 40 °C after one minute. We then removed the pressure and cooled the heating device for one minute at room temperature (23 °C). This process was repeated ten times, and the thermal images at the end of each heating and cooling is given in Supplementary Figure 16a. Supplementary Figure 16b shows the photographs of the copper foil on the surface of the heated film before and after the experiment. There were some slight indentations on the surface of the copper foil but the deformation is minimal. The copper foil did not undergo a severe plastic deformation since the strain of the film was small when activating the heating device (generally less than 5%). Once the external force is removed, the film can be restored to its original state immediately, and the copper foil will also be restored. Therefore, the slight deformation of the copper foil does not affect the repeated use of the heating device.

For the hand-held heating column presented in Fig. 5g, it was first gripped for one minute to generate heat, and then cooled at room temperature for four minutes. We repeated this process for three times. Thermal images of each cycle are shown in Supplementary Figure 16c. As we can see, the initial temperature of the heating column was 24.8 °C. In each cycle, it can be heated to ~45 °C in one minute and cooled to below 30 °C after four minutes, indicating its ability for repeated work.

Supplementary Note 14: Working duration of the hand-held heating column.

To test how long the hand-held heating column presented in Fig. 5g can work with a fully charged lithium ion battery (3.7 V, 1800 mAh). We removed the heating film (including the copper foil and insulating rubber layer) on the side wall of the heating column and unrolled it on a plastic PMMA board. Then we applied a pressure of 200 kPa over a 10 cm^2 of the film and powered the film by a lithium battery (boosted to 28 V by a DC-DC boost module). The current-time curve during this period is shown in Supplementary Figure 17. At the moment of switching on the power, the current passed through the device was 100 mA. After that, the resistance increased due to the temperature rise of the heating film, and the current dropped and stabilized at ~70 mA after 2 minutes. The device temperature at this time was 46.1 °C. The current was maintained at ~70 mA and the device temperature was also stable at ~45 °C for the next three hours. After three hours, the current decreased rapidly and dropped to 35 mA at 4 hours. At this time, the temperature of the device was less than 30 °C. Therefore, this hand-held heating column can work for ~3 hours with such a lithium battery.

Supplementary References

1. Suzuki S., Leinfelder K., Kawai K., Tsuchitani Y. Effect of particle variation on wear rates of posterior composites. *Am. J. Dent.* **8**, 173-178 (1995).

2. Park S., *et al.* Silicones for stretchable and durable soft devices: beyond Sylgard-184. *ACS Appl. Mater. Interfaces* **10**, 11261-11268 (2018).

3. Tutika R., Zhou S. H., Napolitano R. E., Bartlett M. D. Mechanical and Functional Tradeoffs in Multiphase Liquid Metal, Solid Particle Soft Composites. *Adv. Funct. Mater.* **28**, 1804336 (2018).
