# Supplementary material for: Liquid metal-filled magnetorheological elastomer with positive piezoconductivity
Source: Nat Commun. 2019 Mar 21;10:1300. doi: 10.1038/s41467-019-09325-4 (PMC6428896; doi:10.1038/s41467-019-09325-4)
Supplement: Supplementary file 2 — Peer Review File [file 41467_2019_9325_MOESM2_ESM.pdf]

Reviewers' comments:

Reviewer #1 (Remarks to the Author):

In this manuscript, Yun et al. introduces a sample fabrication method to obtain a hybrid composite, which comprises of PDMS (elastomer), liquid metal (soft conductor filler) and iron or nickel nano/microparticles (hard filler). After mixing and solidification, a conductive material was obtained, which showed an increase in conductivity upon compression and stretching, i.e., a positive positive piezoconductivity. Subsequently, this effect is explained and applications of the soft conductive material, such as a hand-held heating column, pressure sensitive heating device and strain sensor. It is one of the most innovative works on liquid metal I have read over the past few months. The work is very well organized and explained. And the result sounds promising and should have significant impacts to the design of composite materials. Besides, the fabrication strategy for the designing of smart materials is simple and useful, which can stimulate the future research toward the design of high-performance composite materials, in particular, the design of emerging liquid metal-based sensors and devices. I would recommend the publication of this work following a minor revision

(1) Since this investigations is based on the liquid metal droplet-filled PDMS. The size of the droplets should have some effects on the performance of the as-made composite. Would the authors could give some comments/explanation on the effects of dimensions of the droplets.

(2) Figure 1e shows that PDMS does not form a continuous phase. It would be better to give some detail explanation/description. The caption of the inset in Figure 1b seems missing. How is the composite looks like? An optical image showing the composite should be provided.

(3) Figure 1: scale bar in 1d and e missing, from the image and the figure caption it is not clear for which size the scale bar is denoting.

(4) Line 100 the degree symbol is missing.

(5) Page 11 line 227, “elongating the LMMRE also increases the surface area of the embedded EGaIn microdroplets”. The EGaIn microdroplets are stretched in the direction of elongating, but the microdroplets may be compressed in the direction perpendicular to the elongating direction. Can the authors provide some schemes or data to illustrate such an interesting morphological change of the droplets during the stretching.

(6) Line 238: “Fig. 3d” The authors probably meant Fig. 2d.

(7) How long can the heating device in Figure 5g work with one battery?

(8) Recently, the authors and several groups have demonstrated methods to the fabrication and applications of liquid metal-droplets at different length scales with different surface properties. I am wondering if the surface properties of the liquid metal droplets play essential roles in the future design of such liquid metal droplet-encapsulated composites. Discussions or explanation with updated references could be helpful to further strengthen the significance of this study.

Reviewer #2 (Remarks to the Author):

In this manuscript, the authors presented a new Liquid Metal-filled Magnetorheological Elastomer with Positive Piezoconductivity for strain, pressure, and magnetic field sensing. Overall the work is valuable, a new functionality is presented with positive piezoconductivity, which is well designed

and demonstrated in detail. However, important details for replicating this work is current missing and a couple major questions remain. Therefore, the reviewer suggests that a series of questions need to be clarified before publishing.

The authors have explored the influence of the mass fraction of Fe particles and liquid metal. Currently, this influence is only studied a single mass fraction of liquid metal or metal particles. There seems to be some advantage to further studying the composite at lower mass fraction of metal particles (Fe or Ni), while increasing the mass fraction of LM.

Due to the nonlinear and inelastic behaviors of soft materials, in addition to the current plots, it would be useful to present the cyclic loading experiments as a function of stain to evaluate the hysteresis of the LMMRE.

On page 10, authors provide a great overview of different types of conductive elastic composites. Within this overview, the authors should also include conductive elastic composites that maintain a constant resistance during stretch:
- https://doi.org/10.1002/adma.201805536
- https://doi.org/10.1038/s41563-018-0084-7
- https://doi.org/10.1002/aelm.201800137
- https://doi.org/10.1002/adma.201706157

Currently the explanation for the change in conductivity during strain is unclear. The current hypothesis: "However, since EGaIn is soft, elongating the LMMRE also increases the surface area of the embedded EGaIn microdroplets; this may enable additional Fe particles to be in contact with the EGaIn droplets during stretching to form additional conductive paths. This could explain the enhancement of the conductivity of the LMMRE during elongation." is confusing, as reported by the authors, the addition of EGaIn results in an increase in resistance, instead of decrease. One would expect that the increase in EGaIn particles would increase the likelihood of contract between the metal fillers. Furthermore, in the presence of oxygen, EGaIn will form an insulating oxide skin. As shown by the Dickey and Kramer groups, films of EGaIn nanoparticles only become electrically conductive after applying localized pressure that merges the particles together to form conductive traces, therefore contact alone with the oxide skin is not enough to form a conductive network.

When measuring the thermal conductivity of the different composites. How did the authors select the mass ratios 1:1.3 and 1:4? Frankly, as currently shown in the SI and Figure 5a and stated within the discussion (Page 22): "Apart from electrical properties, we discovered that the LMMRE also has superior thermal conductivity in comparison to composites with single component fillers." , this is confusing and misleading. This result also contradicts the findings of a recent study by Tutika et al., Adv. Func. Mater., 2018 (https://doi.org/10.1002/adfm.201804336). Furthermore, based on the general conclusion from page 19, first paragraph: "We can see that both the PDMS and PDMS-EGaIn composite have low α, while the LMMRE has the highest α due to the high volume fraction of the metal fillers with large k", this large difference in thermal conductive is likely due the differences in volume fraction of metal fillers. A more direct comparison and informative study that would highlight the LMMRE, would be to compare the different types of composites (PDMS-EGaIn, Fe-MRE, Ni-MRE, Fe-LMMRE, Ni-LMMRE) with equal volume loadings of metal fillers. With the current study, it is unclear if the thermal conductivity and large differences in thermal signatures shown in Figure 5a is simply due the large differences in the volume loading of metal fillers or the type of composite that is presented by the authors.

The functionality presented in Figure 5b-g is quite interesting. Is this process reversible? Or does the copper foil plastically deform when pressure is applied? Cyclic compression experiments would be help to further understand this response.

Overall, the paper is lacking significant details within the methods section. Currently there are no details about how the different composites are fabricated or the measurement techniques used for

characterization. For example, as most multimeters can not register resistances above 10 megaohms, how was the electrical conductivity of the composite measured?

Minor comments:
- Because of the large difference in density, it would be helpful to see the ratios of polymer and metal filler expressed as a volume fraction of the composite, in addition to mass fraction which is currently stated within the manuscript.
- For all cyclic loading experiments, it would be helpful for the authors to present not only the last few loading cycles but also loading cycles at different points within the experiment: beginning, middle and end.
- Does curing temperature have an influence on the resistance of the composite?
- Typo: 30 V voltage (page 20)
- Can the authors expand upon this statement (page 20, first paragraph): "In addition, the simple circuit structure makes it less susceptible to damage." It is currently unclear to the reader how this circuit structure is less susceptible to damage or what this circuit should be compared to.
- Page 23: "The LMMRE can therefore have both the high gauge factor and the low electrical resistance at *high* strains." Currently, all demonstrations are shown at fairly low strains < 20%
- In Figure 4b, what does the initial rise in resistance from approximately 0 to 10 seconds correspond to?
- "As a control, we fabricated a composite only containing PDMS (1 g) and Ni particles (2 g) without using EGaIn, and found that this composite is basically an insulator under compressive strains small than –0.05." What does basically an insulator refer to?
- Page 20: "Due to the positive piezoconductive characteristic of the LMMRE, this heating film can also generate heat during stretching when applying a current." The composite can not generate heat but is capable of *producing* heat based on the Joule heating effect.

Reviewer #1

Reviewer #1: In this manuscript, Yun et al. introduces a sample fabrication method to obtain a hybrid composite, which comprises of PDMS (elastomer), liquid metal (soft conductor filler) and iron or nickel nano/microparticles (hard filler). After mixing and solidification, a conductive material was obtained, which showed an increase in conductivity upon compression and stretching, i.e., a positive piezoconductivity. Subsequently, this effect is explained and applications of the soft conductive material, such as a hand-held heating column, pressure sensitive heating device and strain sensor. It is one of the most innovative works on liquid metal I have read over the past few months. The work is very well organized and explained. And the result sounds promising and should have significant impacts to the design of composite materials. Besides, the fabrication strategy for the designing of smart materials is simple and useful, which can stimulate the future research toward the design of high-performance composite materials, in particular, the design of emerging liquid metal-based sensors and devices. I would recommend the publication of this work following a minor revision:

We thank the reviewer for such a positive comment.

1. Since this investigations is based on the liquid metal droplet-filled PDMS. The size of the droplets should have some effects on the performance of the as-made composite. Would the authors could give some comments/explanation on the effects of dimensions of the droplets.

Per the reviewer's comment, we have conducted additional experiments to analyse the influence of EGaIn droplet size on the electrical properties of the LMMRE, as given in the Supplementary Information S2. We also added the following descriptions into the main manuscript:

Page 6, Line 24

In addition to the content, the size of the EGaIn droplets also has a great influence on the electrical properties of the composite. We prepared a batch of LMMRE samples with different EGaIn droplet diameters by controlling the rotating speed of the electric stirrer (see Supplementary Information S2 for details). Our results show that the LMMRE with smaller EGaIn droplets (< 10 μm) has a higher resistivity because a smaller EGaIn droplet has fewer adjacent Fe particles and forms fewer conductive paths (cf. Fig. S2c). However, large EGaIn droplets (> 25 μm) can reduce the mechanical strength and the stability of the resistance measurement (indicated by the larger error bars in Fig. S2c). Therefore, we controlled the droplet diameter to about 15 μm in the subsequent experiments to achieve both the low resistivity and the high stability.

Supplementary Information S2

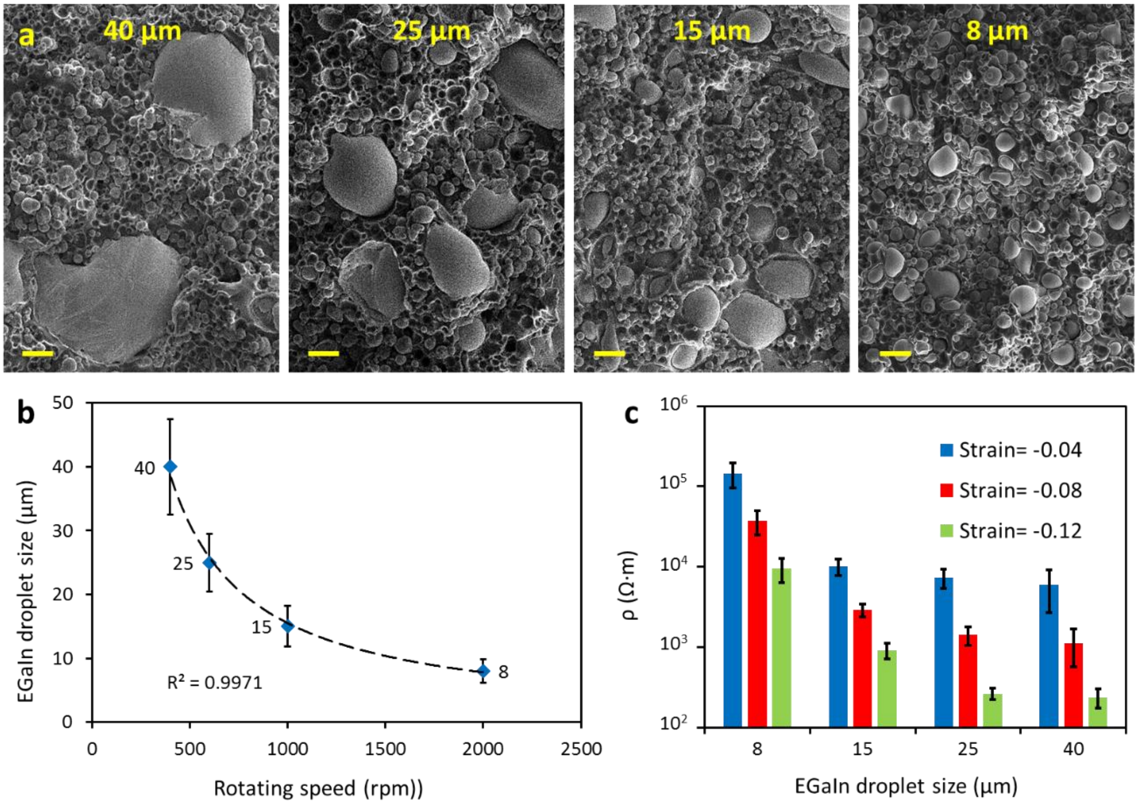

Figure S2. Influence of EGaIn droplet size on the electrical properties of LMMRE. a SEM images of LMMRE samples with different EGaIn droplet diameters. Scale bars are 10 µm. **b** Average diameter of EGaIn droplets in LMMRE *vs* stirring speed. **c** Resistivity of the LMMRE with different EGaIn droplet diameters.

We prepared four LMMRE samples using 1 g PDMS, 4 g iron powder and 1.25 g EGaIn (PDMS/Fe particle/EGaIn volume ratio of 1:0.51:0.2). To analyse the influence of EGaIn droplet size on the electrical properties of LMMRE, we mixed the raw materials under different rotating speeds of the electric stirrer to control the EGaIn droplet size within the LMMRE. Due to the large surface tension of EGaIn, a large shear force is required during the stirring process to break it into microdroplets. The shearing force depends on many factors such as the stirring speed, the size of the stirring head and the viscosity of the liquid. Due to the same ratio of raw materials, the viscosity of the mixed liquids of the four samples is basically the same. We used a cylindrical plastic stick (diameter of 4 mm) as the stirrer and stirred the mixture for 5 minutes at different rotating speeds (400, 600, 1000, 2000 rpm, respectively). We measured the size of the EGaIn droplets in the four LMMRE samples (Fig. S2a) and obtained their relationship to the rotating speed (Fig. S2b). Obviously, the diameter of the EGaIn droplet is inversely proportional to the rotating speed (R^2 = 0.9971). This is because the droplet size is inversely proportional to the shear force, which is proportional to the linear velocity at the edge of the stirring head. Since the diameter of the stirring head is constant, the line speed is proportional to the rotating speed.

Fig. S2c shows the resistivity of these LMMRE samples. As we can see, the LMMRE with smaller EGaIn droplets has higher resistivity. In LMMRE, the EGaIn droplets can connect adjacent Fe particles

and form conductive pathways to improve the conductivity of the composite. This phenomenon is especially significant when the EGaIn droplet is much larger than the Fe particles (diameter 2-5 μm). If the diameter of the EGaIn droplets is too small (< 10 μm), the amount of Fe particles that it can connect will decrease drastically, resulting in a rapid rise in the resistivity of the composite. However, large droplets create large cavities in the composite that are easily broken under mechanical deformation and cause EGaIn to seep out. This impairs the mechanical strength and the resistivity stability of the composite (indicated by the larger error bars in Fig. S2b). Therefore, we choose the stirring speed of 1000 rpm in the subsequent sample fabrication to achieve both the low resistivity and the high stability of measurement. In this case, the EGaIn droplet diameter is about 15 μm.

2. Figure 1e shows that PDMS does not form a continuous phase. It would be better to give some detail explanation/description. The caption of the inset in Figure 1b seems missing. How is the composite looks like? An optical image showing the composite should be provided.

Per the reviewer's comment, we added the following description into the main manuscript to explain the discontinuous distribution of silicon in the EDS image of Figure 1e, as given below:

Page 5, Line 17

Since the EDS analysis only scans the elements on the surface layer of the material, the PDMS matrix is obscured by Fe particles, EGaIn droplets, and their shadows, resulting in a seemingly discontinuous distribution of Si elements. As can be seen from the SEM images, the PDMS matrix is actually continuously distributed.

We also added the caption of the inset in the description of Figure 1b, as given below:

Page 10, Line 2

b SEM images of the obtained LMMRE. The inset shows a micrograph of a LMMRE sample.

Per the reviewer's comment, we provided an optical image of the cross section of the composite in the Supplementary Information S1, as given below:

Supplementary Information S1

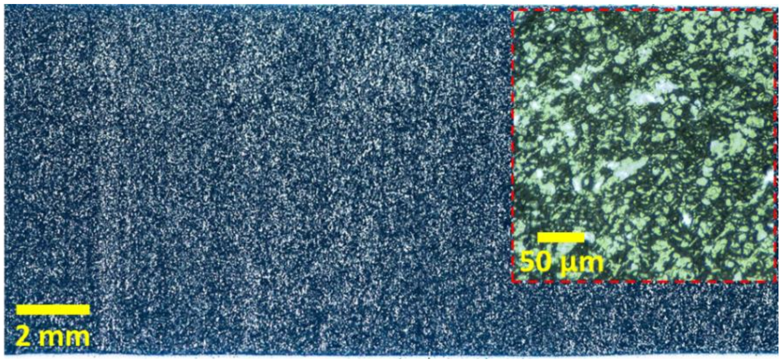

Figure S1. Optical image of the LMMRE. Optical images of the LMMRE obtained using a digital SLR camera and an optical microscope. The white spots in the image are EGaIn droplets. The overall colour of the LMMRE is silver grey.

3. Figure 1: scale bar in 1d and e missing, from the image and the figure caption it is not clear for which size the scale bar is denoting.
Per the reviewer's comment, we added the scale bars and their size in Figure 1, as given below:<br>***Page 10, Line 1***<br>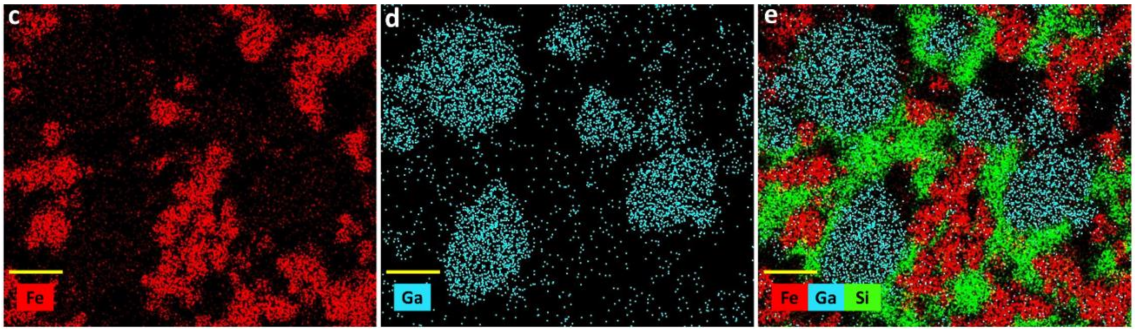<br><br>**Fig 1 c-e** EDS element mappings of the LMMRE. Scale bars are 10 μm.
4. Line 100 the degree symbol is missing.
Per the reviewer's comment, we added the degree symbol, as given below:<br>***Page 5, Line 6***<br>Finally, we placed the mixture in an oven and cured it at 70 ℃ for 6 hours to obtain the composite.
5. Page 11 line 227, "elongating the LMMRE also increases the surface area of the embedded EGaIn microdroplets". The EGaIn microdroplets are stretched in the direction of elongating, but the microdroplets may be compressed in the direction perpendicular to the elongating direction. Can the authors provide some schemes or data to illustrate such an interesting morphological change of the droplets during the stretching?
Under stretching, the liquid metal droplet has a constant volume and will change from spherical to ellipsoidal shape. For a given volume, the object with the smallest surface area is the sphere, therefore the surface area of the EGaIn microdroplet increases under stretching. However, we calculated that the surface area of the droplets only increases by about 0.8% at 20% tensile strain, which somehow cannot explain the sharp decrease in the LMMRE resistivity during stretching. Therefore, we modified our explanation of the dramatic reduction of the LMMRE's resistivity during mechanical deformation, and we verified it using numerical simulation, as given in Supplementary Information S7. We also added the following descriptions into the main manuscript:<br>***Page 12, Line 16***<br>Since the resistivity of the PDMS is much higher than that of iron, EGaIn, and the thin gallium oxide layer, we believe the resistivity of the LMMRE mainly depends on the thickness of the PDMS matrix along the conductive path. Due to the constant volume (the Poisson's ratio of ~0.5), the LMMRE will always be compressed in a certain direction upon any mechanical deformation. During stretching, the EGaIn droplets deform along with the PDMS matrix, but the rigid Fe particles do not. Consequently, the Fe particle may squeeze the surrounding PDMS matrix and EGaIn droplets, leading to a sharp reduction in the thickness of the PDMS layer between them, and even enable additional Fe particles to be in direct

contact with the EGaIn droplets. We believe this effect would provide more conductive paths to reduce the overall resistivity of the composite during stretching. To verify this hypothesis, we constructed a 2D model of the LMMRE based on the SEM images and the calculated Poisson's ratio; we simulated its resistivity before and after stretching using COMSOL, as shown in Supplementary Information S7. Our simulation results show the reduction in the thickness of the PDMS layer between Fe particles and the EGaIn droplets upon stretching and consequently lead to a significant decrease in the overall resistivity (see Supplementary Information S7 for detailed discussion). Owing to the elasticity of PDMS, the EGaIn microdroplets restore to their original shapes upon removing the external load.

Supplementary Information S7

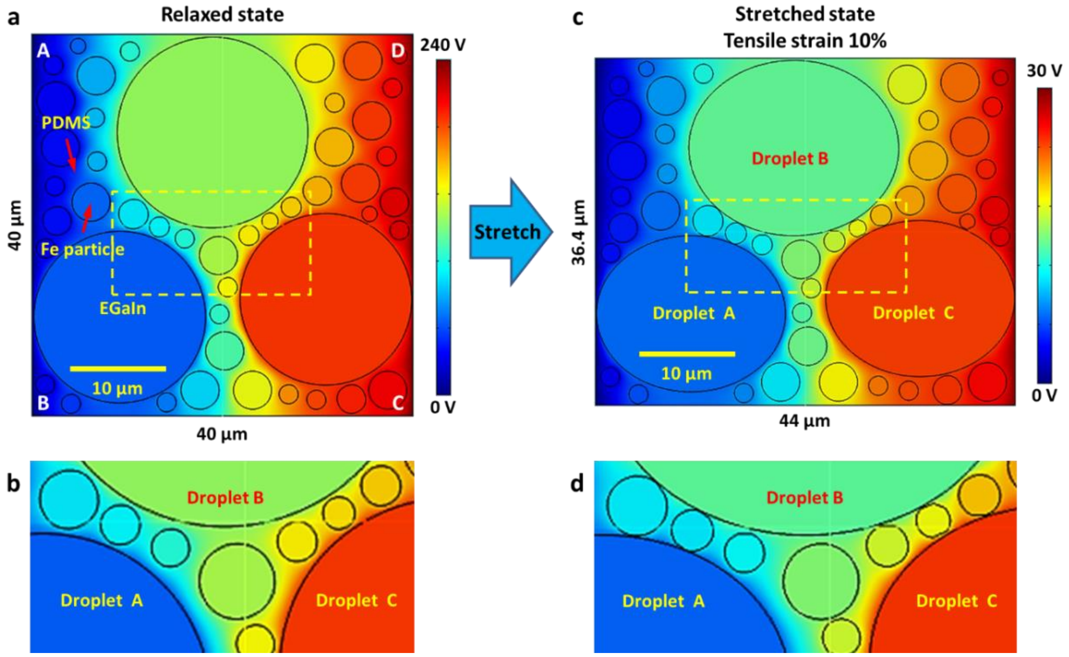

Figure S7. Numerical simulation of the sheet resistance of the LMMRE before and after stretching. Simulation results of the electrical potential distribution of the LMMRE when passing through a constant electrical current density of 125 A/m^2 **a** before and **c** after stretching. **b** and **d** show the enlarged view of the yellow-dashed area in **a** and **c**, respectively.

To simulate the change of resistivity before and after stretching, we drew a 40 × 40 μm LMMRE 2D plane model based on the obtained SEM image and import it into COMSOL for simulation. The Poisson's ratio of the PDMS matrix was set at 0.5, the Fe particles were treated as rigid circles, and the volume of the liquid metal droplets remained constant during stretching. After stretching, the amount of longitudinal elongation of the PDMS and EGaIn droplets in the model equals their transversal compression ratio. EGaIn droplets change from circular to oval shape. Since the Young's modulus of Fe particles is much larger than that of PDMS matrix, Fe particles are spherical before and after stretching, but its centroid position changes along with the PDMS matrix. In the simulation, the AB side of the composite is set to ground and the current density that passes through the CD side is set constant at 125 A/m^2, whereas no current flows through the BC and AD sides. According to the simulation results, the sheet resistance of the LMMRE before and after stretching was 23.99 GΩ/sq and 2.451 GΩ/sq,

respectively. The reason for the sharp drop in sheet resistance may be found in the voltage distribution of LMMRE before and after stretching, as given in Fig. S7. Since the resistivity of the PDMS matrix is much higher than that of iron, EGaIn and its thin surface oxide layer, the resistivity of the composite depends on the thickness of the PDMS matrix on the conductive path. As shown in Fig. S7b, in the relaxed state, there is a thicker PDMS barrier between EGaIn droplet A, B and the Fe particles between them. The potential difference between droplets A and B is ~100 V. When the sample is stretched in the longitudinal direction, it is compressed in the lateral direction, and the distance between the droplets A and B reduces (Fig. S7d). Since the Fe particles maintain a spherical shape and constant diameter during the stretching process, they will squeeze the EGaIn droplets A and B. This may result in a sharp decrease in the thickness of the PDMS layer between the droplets A, B and the Fe particles (see Figs. S7b and d). At this time, the potential difference between the droplets A and B is only ~10 V, and the overall sheet resistance of the composite also reduces greatly. In 3D composites, this phenomenon of reduced resistance will be more obvious.

In principle, no matter what kind of mechanical deformation the LMMRE composite is subjected to, it will always maintain a constant volume (the Poisson's ratio of ~0.5), and be compressed in a certain direction. Although the PDMS thickness between the metal fillers increases slightly along the stretching direction, the distance between the EGaIn droplets and the metal fillers in the transversal and direction is drastically reduced because of the undeformed Fe particles. We believe this reduces the thickness of the PDMS layer along the conductive path and the overall resistivity of the composite. In our future work, we will conduct further experimental investigation using high-resolution 3D scanning instrument such as X-ray computed tomography facility (e.g. nano-CT) to fully verify the change of the microstructures of the LMMRE during deformation.

6. Line 238: “Fig. 3d” The authors probably meant Fig. 2d.

Thank you. Yes, it should be “Fig. 2d”. We have fixed this mistake.

7. How long can the heating device in Figure 5g work with one battery?

Per the reviewer’s comment, we conducted an experiment to test the working time of this heating device, as shown in the Supplementary Information S17:

Supplementary Information S17

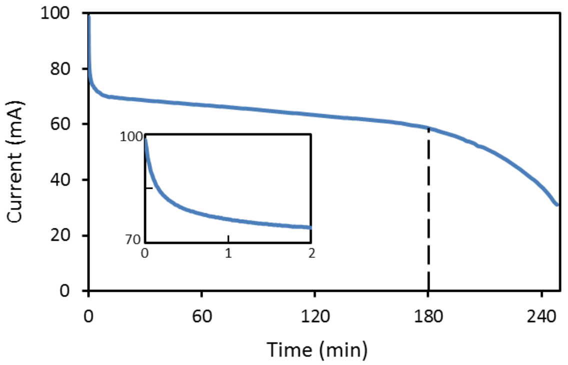

Figure S17. Working duration of the hand-held heating column. Current *vs* time curve of the hand-held heating column powered by a lithium battery (1800 mAh). The inset shows the current-time curve for the first two minutes.

To test how long the hand-held heating column presented in Fig. 5g can work with a fully charged lithium ion battery (3.7 V, 1800 mAh). We removed the heating film (including the copper foil and insulating rubber layer) on the side wall of the heating column and unrolled it on a plastic PMMA board. Then we applied a pressure of 200 kPa over a 10 cm^2 of the film and powered the film by a lithium battery (boosted to 28 V by a DC-DC boost module). The current-time curve during this period is shown in Fig. S17. At the moment of switching on the power, the current passed through the device was 100 mA. After that, the resistance increased due to the temperature rise of the heating film, and the current dropped and stabilized at ~70 mA after 2 minutes. The device temperature at this time was 46.1 ℃. The current was maintained at ~70 mA and the device temperature was also stable at ~45 ℃ for the next three hours. After three hours, the current decreased rapidly and dropped to 35 mA at 4 hours. At this time, the temperature of the device was less than 30 ℃. Therefore, this hand-held heating column can work for ~3 hours with such a lithium battery.

8. Recently, the authors and several groups have demonstrated methods to the fabrication and applications of liquid metal-droplets at different length scales with different surface properties. I am wondering if the surface properties of the liquid metal droplets play essential roles in the future design of such liquid metal droplet-encapsulated composites. Discussions or explanation with updated references could be helpful to further strengthen the significance of this study.

Per the reviewer's comment, we added the following description and references in the main manuscript:

Page 25, Line 13

Apart from investigating the effects of the solid microparticle fillers, modifying the surface properties of liquid metal, such as coating it with a layer of semiconductive or conductive nanoparticles[35-37], may also affect the overall electrical properties of the composite.

35. Chen Y., et al. Robust Fabrication of Nonstick, Noncorrosive, Conductive Graphene-Coated Liquid Metal Droplets for Droplet-Based, Floating Electrodes. *Advanced Functional Materials* **28**, 1706277 (2018).

36. Sivan V., et al. Liquid metal marbles. *Advanced Functional Materials* **23**, 144-152 (2013).

37. Carey B. J., et al. Wafer-scale two-dimensional semiconductors from printed oxide skin of liquid metals. *Nature Communications* **8**, 14482 (2017).

**Reviewer #2:**
In this manuscript, the authors presented a new Liquid Metal-filled Magnetorheological Elastomer with Positive Piezoconductivity for strain, pressure, and magnetic field sensing. Overall the work is valuable, a new functionality is presented with positive piezoconductivity, which is well designed and demonstrated in detail. However, important details for replicating this work is current missing and a couple major questions remain. Therefore, the reviewer suggests that a series of questions need to be clarified before publishing.
We thank the reviewer for the overall positive comment.
1. The authors have explored the influence of the mass fraction of Fe particles and liquid metal. Currently, this influence is only studied a single mass fraction of liquid metal or metal particles. There seems to be some advantage to further studying the composite at lower mass fraction of metal particles (Fe or Ni), while increasing the mass fraction of LM.
Per the reviewer's comment, we conducted additional experiments by fabricating four different LMMREs and compared their electrical properties. In these composites, the Fe particle and EGaIn have different mass fractions but the sum of their mass fractions is all 84%. The details of this experiment are given in Supplementary Information S5. We also added the following description into the main manuscript:<br>***Page 8, Line 19***<br>To further analyse the influence of the relative content of Fe particle and EGaIn on the electrical properties of the composite, we prepared four LMMRE samples and compared their electrical properties. The Fe particle and EGaIn in these composites have different contents, but the sum of their mass fractions is all 84% (see Supplementary Information S5 for details). We can see that LMMRE containing 64% Fe particles and 20% EGaIn (i.e. 1 g PDMS, 4 g Fe particles and 1.25 g EGaIn) has the lowest resistivity (cf. Fig. S5b), which is consistent with our previous conclusions.<br><br>**Supplementary Information S5: Resistivity of the LMMRE prepared using different Fe particle and EGaIn contents**<br>We prepared four LMMRE samples using 1 g PDMS and 5.25 g metal filler (including iron powder and EGaIn). The qualities and mass fractions of raw materials contained in these samples are given in Table S1. The SEM images (Fig. S5a) show the large difference in EGaIn content in Samples A and D. We measured the resistivity-strain curves of the four samples, as shown in Fig. S5b, in which we can see that as the content of Fe particles decreased and the content of EGaIn increased, the resistivity of the samples rose sharply. Sample D was always electrically insulated at any strain and would break when the compressive strain exceeds 60%.

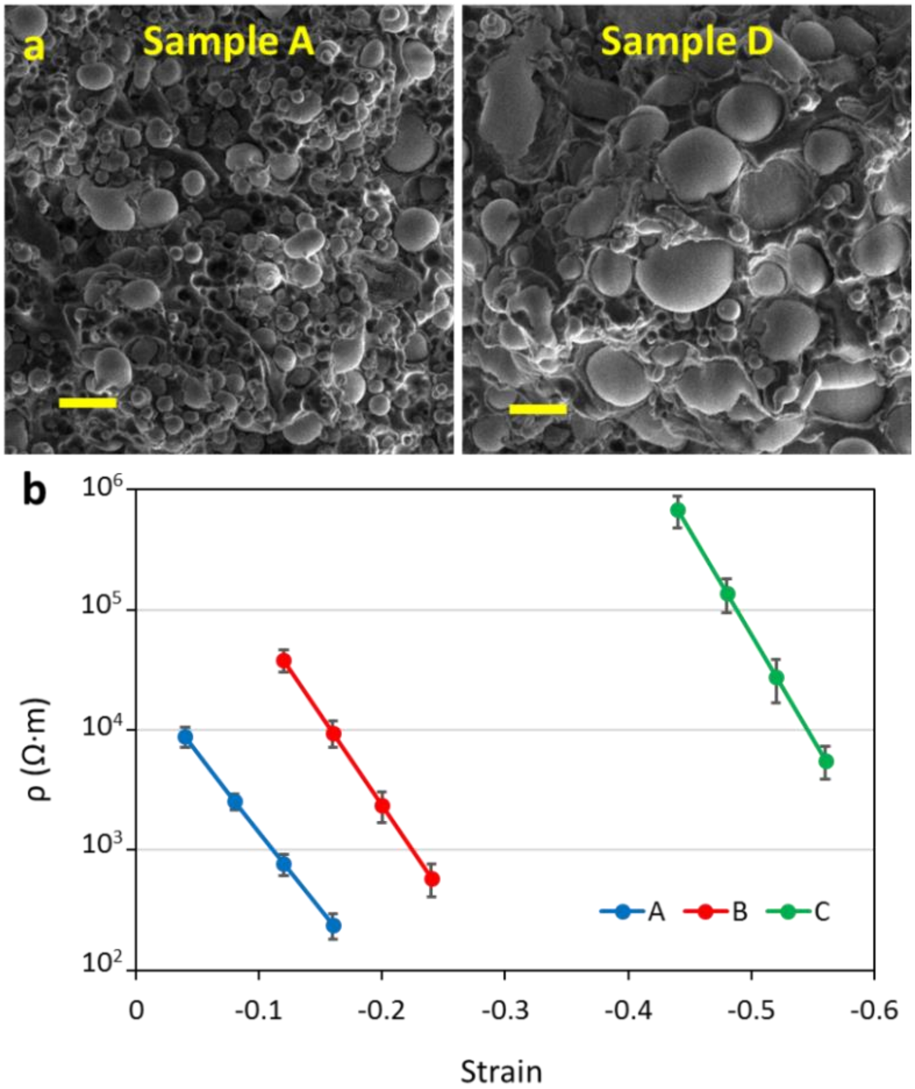

Figure S5. a SEM images of Samples A and D. Scale cars are 10 μm. **b** Resistivity of the Fe-LMMRE prepared with different iron particle and EGaIn contents *vs* compressive strain.

Table S1. Qualities and mass fractions of raw materials contained in 4 samples.

Sample	PDMS	Fe particles	EGaIn
Sample A	1 g (16%)	4.0 g (64%)	1.25 g (20%)
Sample B	1 g (16%)	3.5 g (56%)	1.75 g (28%)
Sample C	1 g (16%)	3.0 g (48%)	2.25 g (36%)
Sample D	1 g (16%)	2.5 g (40%)	2.75 g (44%)

2. Due to the nonlinear and inelastic behaviors of soft materials, in addition to the current plots, it would be useful to present the cyclic loading experiments as a function of stain to evaluate the hysteresis of the LMMRE.

Per the reviewer's comments, we performed additional cyclic loading-unloading experiments on the LMMRE and provided the stress-strain curves in Supplementary Information S10. We also added the following descriptions into the main manuscript:

Page 14, Line 10

In addition, we also performed the cyclic loading-unloading experiments on the LMMRE and obtained the cyclic compressive and tensile stress-strain curves, as given in Supplementary Information S10. We can see that LMMRE exhibits an obvious elastic hysteresis (cf. Fig. S10). However, the elastic hysteresis of LMMRE has no significant effect on its electrical properties. According to the resistance change curves under cyclic loading in Fig. 2, the resistance change of LMMRE during the loading and unloading process is basically symmetrical. Furthermore, the LMMRE also shows excellent cycle stability, which is beneficial to its repeated use.

Supplementary Information S10

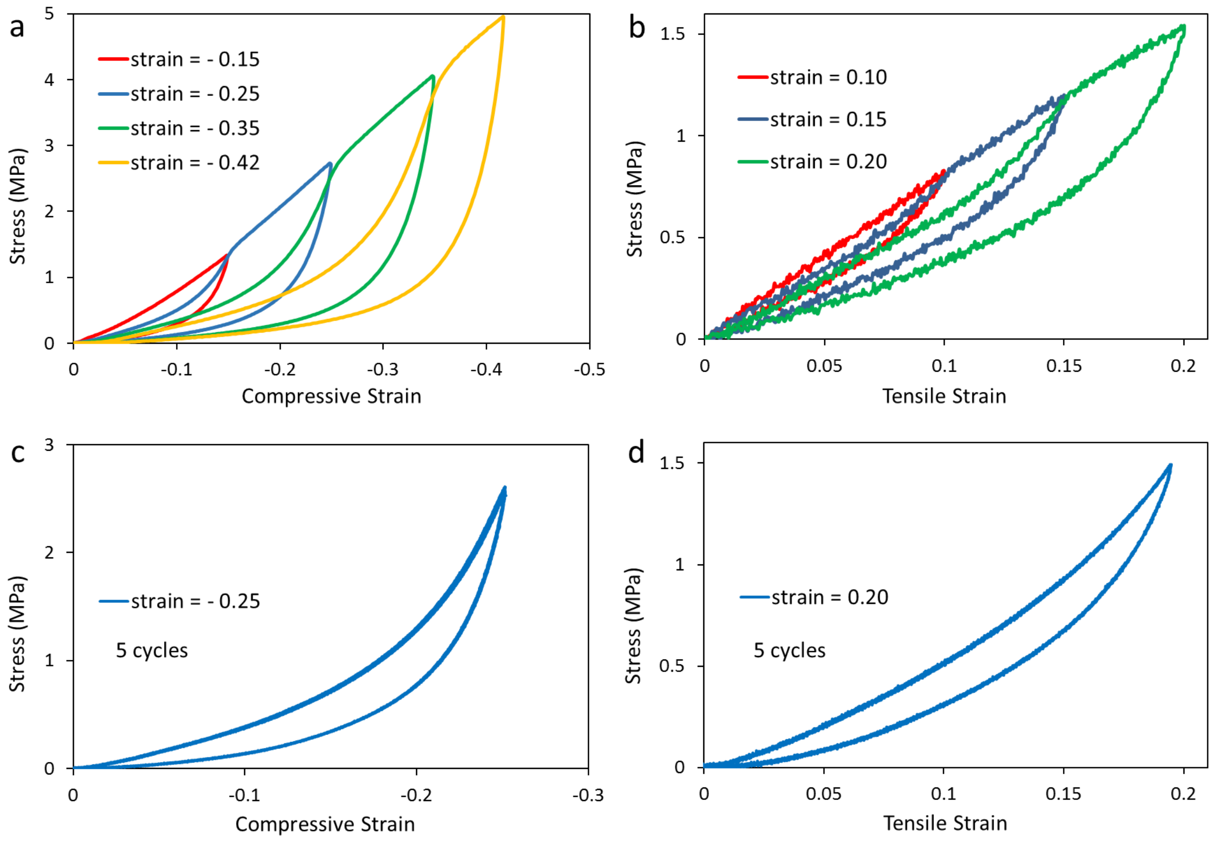

Figure S10. Cyclic loading–unloading tests of the LMMRE. Cyclic **a** compressive and **b** tensile loading–unloading plots of LMMRE with increasing strains. Cyclic **c** compressive and **d** tensile loading–unloading plots of LMMRE to the same maximum strain.

In the cyclic loading-unloading experiments, we applied a cyclic load to the LMMRE sample using the MTS Landmark 370.02 hydraulic load frame to measure its stress-strain behaviour. In Figs. S10a and S10b, the maximum load for each cycle is gradually increased, while the maximum load for each cycle in Figs. S10c and S10d remained constant. We can see that the LMMRE exhibits obvious elastic hysteresis. Besides, the Young's modulus of the LMMRE in the relaxed state decreases as the maximum load increases. Under the same maximum load, the stress-strain curves of LMMRE in 5 cycles are completely coincident (Figs. S10c and S10d), indicating that the LMMRE has an excellent cycle stability.

3. On page 10, authors provide a great overview of different types of conductive elastic composites. Within this overview, the authors should also include conductive elastic composites that maintain a constant resistance during stretch:

- https://doi.org/10.1002/adma.201805536
- https://doi.org/10.1038/s41563-018-0084-7
- https://doi.org/10.1002/aelm.201800137
- https://doi.org/10.1002/adma.201706157

Per the reviewer's comment, we carefully studied these works and added them into the reference of the main manuscript, we also added the following descriptions into the main manuscript:

Page 3, Line 18

In addition, recent studies also demonstrated liquid metal-filled elastic composites that can minimise the reduction of resistance during stretching[24-27].

Page 11, Line 10

Using the fluidity and high electrical conductivity of liquid metals, a few recent studies developed liquid metal-filled conductive elastomeric composites that can minimise the reduction of resistance during stretching. Some of them have electrically self-healing capabilities. When stretched, the oxide film of the liquid metal droplets in these composites will rupture. The liquid metal can flow out and form new connections with adjacent solid conductive fillers to retain the overall resistance of the composite[24-26]. In another composite material, liquid metal droplets can form a 3D Calabash Bunch conductive network structure in the elastic matrix to maintain the resistance during extension[27].

24. Wang J., Cai G., Li S., Gao D., Xiong J., Lee P. S. Printable superelastic conductors with extreme stretchability and robust cycling endurance enabled by liquid-metal particles. *Advanced Materials* **30**, 1706157 (2018).

25. Park S., Thangavel G., Parida K., Li S., Lee P. S. A Stretchable and Self-Healing Energy Storage Device Based on Mechanically and Electrically Restorative Liquid-Metal Particles and Carboxylated Polyurethane Composites. *Advanced Materials* **31** 1805536 (2018).

26. Markvicka E. J., Bartlett M. D., Huang X., Majidi C. An autonomously electrically self-healing liquid metal–elastomer composite for robust soft-matter robotics and electronics. *Nature Materials* 17,

618-624 (2018).

27. Yu Z., et al. A Composite Elastic Conductor with High Dynamic Stability Based on 3D-Calabash Bunch Conductive Network Structure for Wearable Devices. *Advanced Electronic Materials* **4**, 1800137 (2018).

4. Currently the explanation for the change in conductivity during strain is unclear. The current hypothesis: "However, since EGaIn is soft, elongating the LMMRE also increases the surface area of the embedded EGaIn microdroplets; this may enable additional Fe particles to be in contact with the EGaIn droplets during stretching to form additional conductive paths. This could explain the enhancement of the conductivity of the LMMRE during elongation." is confusing, as reported by the authors, the addition of EGaIn results in an increase in resistance, instead of decrease. One would expect that the increase in EGaIn particles would increase the likelihood of contract between the metal fillers. Furthermore, in the presence of oxygen, EGaIn will form an insulating oxide skin. As shown by the Dickey and Kramer groups, films of EGaIn nanoparticles only become electrically conductive after applying localized pressure that merges the particles together to form conductive traces, therefore contact alone with the oxide skin is not enough to form a conductive network.

We thank the reviewer for the comment. Per the reviewer's comment, we revised our explanation for the dramatic reduction of the LMMRE's resistivity during mechanical deformation, and verified our hypothesis using numerical simulation, as detailed in Supplementary Information S7. Since the resistivity of PDMS is much higher than that of the thin gallium oxide layer on the surface of EGaIn droplets, we believe the overall resistivity of the LMMRE mainly depends on the thickness of the PDMS matrix between the Fe particles and the EGaIn droplets. Such a PDMS layer is much thicker than the thin gallium oxide layer (few nm). Under mechanical deformation, the thickness of the PDMS between metal fillers is drastically decreased and therefore, the overall resistivity of the LMMRE composite can be reduced.

Moreover, we expect that the effect of the oxide layer is more pronounced if the liquid metal droplets are nanosized such that a large number of liquid metal nanoparticles can result in a large overall thickness of the oxide layer. This makes the liquid metal nanoparticle network electrically insulated before breaking the oxide layer, as reported in Dickey and Kramer's works (e.g. Small, 2015, 11, 6397). We believe that in our case the gallium oxide layer has less effect on the composite's overall resistivity since the size of the droplets in the LMMRE is large (tens of µm). To clarify this confusion, we added the following descriptions into the main manuscript:

Page 12, Line 16

Since the resistivity of the PDMS is much higher than that of iron, EGaIn, and the thin gallium oxide layer, we believe the resistivity of the LMMRE mainly depends on the thickness of the PDMS matrix along the conductive path. Due to the constant volume (the Poisson's ratio of ~0.5), the LMMRE will always be compressed in a certain direction upon any mechanical deformation. During stretching, the EGaIn droplets deform along with the PDMS matrix, but the rigid Fe particles do not. Consequently, the Fe particle may squeeze the surrounding PDMS matrix and EGaIn droplets, leading to a sharp reduction in the thickness of the PDMS layer between them, and even enable additional Fe particles to be in direct contact with the EGaIn droplets. We believe this effect would provide more conductive paths to reduce the overall resistivity of the composite during stretching. To verify this hypothesis, we constructed a 2D model of the LMMRE based on the SEM images and the calculated Poisson's ratio; we simulated its resistivity before and after stretching using COMSOL, as shown in Supplementary Information S7. Our

simulation results show the reduction in the thickness of the PDMS layer between Fe particles and the EGaIn droplets upon stretching and consequently lead to a significant decrease in the overall resistivity (see Supplementary Information S7 for detailed discussion). Owing to the elasticity of PDMS, the EGaIn microdroplets restore to their original shapes upon removing the external load.

Supplementary Information S7

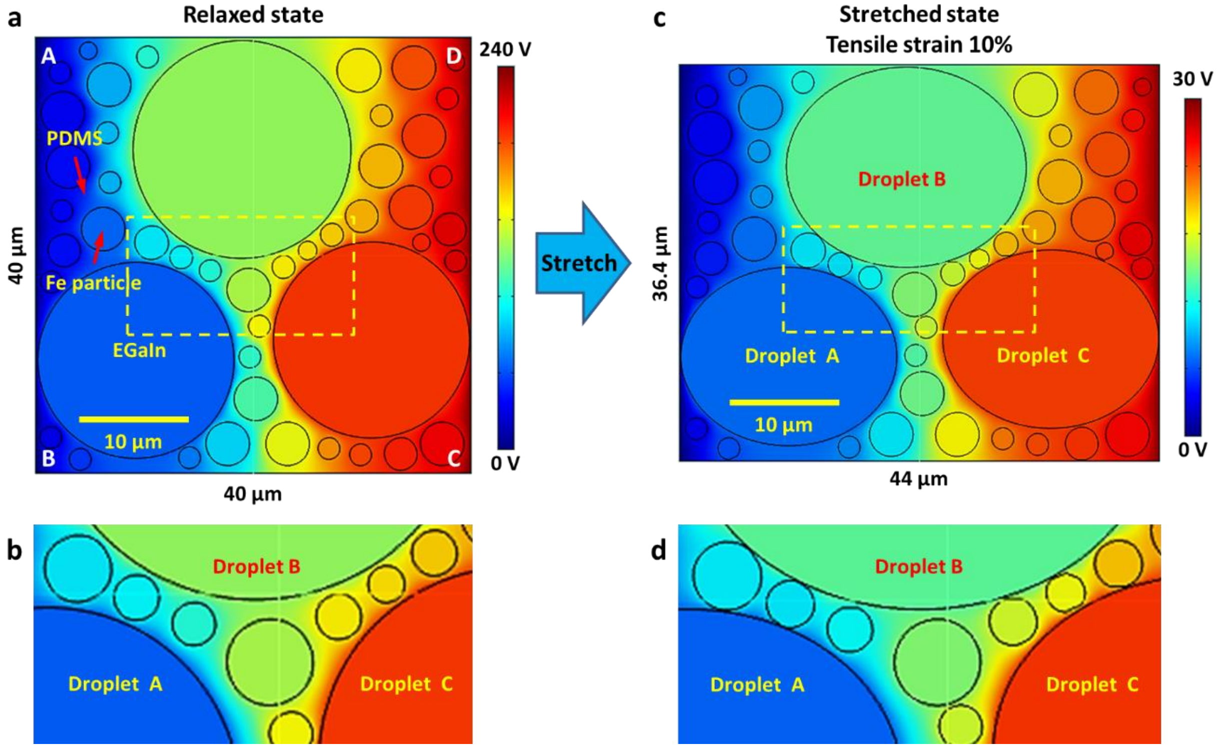

Figure S7. Numerical simulation of the sheet resistance of the LMMRE before and after stretching. Simulation results of the electrical potential distribution of the LMMRE when passing through a constant electrical current density of 125 A/m^2 **a** before and **c** after stretching. **b** and **d** show the enlarged view of the yellow-dashed area in **a** and **c**, respectively.

To simulate the change of resistivity before and after stretching, we drew a 40 × 40 µm LMMRE 2D plane model based on the obtained SEM image and import it into COMSOL for simulation. The Poisson's ratio of the PDMS matrix was set at 0.5, the Fe particles were treated as rigid circles, and the volume of the liquid metal droplets remained constant during stretching. After stretching, the amount of longitudinal elongation of the PDMS and EGaIn droplets in the model equals their transversal compression ratio. EGaIn droplets change from circular to oval shape. Since the Young's modulus of Fe particles is much larger than that of PDMS matrix, Fe particles are spherical before and after stretching, but its centroid position changes along with the PDMS matrix. In the simulation, the AB side of the composite is set to ground and the current density that passes through the CD side is set constant at 125 A/m^2, whereas no current flows through the BC and AD sides. According to the simulation results, the sheet resistance of the LMMRE before and after stretching was 23.99 GΩ/sq and 2.451 GΩ/sq, respectively. The reason for the sharp drop in sheet resistance may be found in the voltage distribution of LMMRE before and after stretching, as given in Fig. S7. Since the resistivity of the PDMS matrix is

much higher than that of iron, EGaIn and its thin surface oxide layer, the resistivity of the composite depends on the thickness of the PDMS matrix on the conductive path. As shown in Fig. S7b, in the relaxed state, there is a thicker PDMS barrier between EGaIn droplet A, B and the Fe particles between them. The potential difference between droplets A and B is ~100 V. When the sample is stretched in the longitudinal direction, it is compressed in the lateral direction, and the distance between the droplets A and B reduces (Fig. S7d). Since the Fe particles maintain a spherical shape and constant diameter during the stretching process, they will squeeze the EGaIn droplets A and B. This may result in a sharp decrease in the thickness of the PDMS layer between the droplets A, B and the Fe particles (see Figs. S7b and d). At this time, the potential difference between the droplets A and B is only ~10 V, and the overall sheet resistance of the composite also reduces greatly. In 3D composites, this phenomenon of reduced resistance will be more obvious.

In principle, no matter what kind of mechanical deformation the LMMRE composite is subjected to, it will always maintain a constant volume (the Poisson's ratio of ~0.5), and be compressed in a certain direction. Although the PDMS thickness between the metal fillers increases slightly along the stretching direction, the distance between the EGaIn droplets and the metal fillers in the transversal and direction is drastically reduced because of the undeformed Fe particles. We believe this reduces the thickness of the PDMS layer along the conductive path and the overall resistivity of the composite. In our future work, we will conduct further experimental investigation using high-resolution 3D scanning instrument such as X-ray computed tomography facility (e.g. nano-CT) to fully verify the change of the microstructures of the LMMRE during deformation.

5. When measuring the thermal conductivity of the different composites. How did the authors select the mass ratios 1:1.3 and 1:4? Frankly, as currently shown in the SI and Figure 5a and stated within the discussion (Page 22): "Apart from electrical properties, we discovered that the LMMRE also has superior thermal conductivity in comparison to composites with single component fillers." , this is confusing and misleading. This result also contradicts the findings of a recent study by Tutika et al., Adv. Func. Mater., 2018 (https://doi.org/10.1002/adfm.201804336). Furthermore, based on the general conclusion from page 19, first paragraph: "We can see that both the PDMS and PDMS-EGaIn composite have low α, while the LMMRE has the highest α due to the high volume fraction of the metal fillers with large k", this large difference in thermal conductive is likely due the differences in volume fraction of metal fillers. A more direct comparison and informative study that would highlight the LMMRE, would be to compare the different types of composites (PDMS-EGaIn, Fe-MRE, Ni-MRE, Fe-LMMRE, Ni-LMMRE) with equal volume loadings of metal fillers. With the current study, it is unclear if the thermal conductivity and large differences in thermal signatures shown in Figure 5a is simply due the large differences in the volume loading of metal fillers or the type of composite that is presented by the authors.

We thank the reviewer for the comment. Per the reviewer's comment, we conducted additional experiments to compare the thermal conductivity of five different composites (PDMS-EGaIn, Fe-/Ni-MRE, Fe-/Ni-LMMRE). These composites have the same volume fraction of metal fillers. The experimental results and analysis are given in Supplementary Information S14 and S15. We also revised Fig. 5a and the descriptions in the main manuscript, as given below:

Page 20, Line 5

Along with the high sensitivity to strain and magnetic field, the Fe- and Ni-LMMRE developed in this work also have good thermal conductivity. We compared the thermal diffusivity α of the Fe-/Ni-

LMMRE (PDMS/Fe(Ni) particle/EGaIn volume ratio of 1:0.25:0.2) with a mixture of PDMS and EGaIn (PDMS-EGaIn, PDMS/EGaIn volume ratio of 1:0.45), a mixture of PDMS and Fe microparticles (Fe-MRE, PDMS/Fe particle volume ratio of 1:0.45), and a mixture of PDMS and Ni microparticles (Ni-MRE, PDMS/Ni particle volume ratio of 1:0.45). According to the recent study by Tutika et al., the volume fraction of metal fillers in composites has a great effect on the thermal conductivity of the material[34]. To reflect the difference in thermal conductivity of different types of composites, we set the volume ratio of PDMS matrix and metal fillers of the five composites to 1:0.45. The experimental setup is shown in Supplementary Information S13. The initial temperature of the samples was 23 ℃ and the temperature of the plate was maintained at 50 ℃. All of these samples were cylindrical and equal in diameter (10 mm) and height (23 mm). We monitored their temperature change using an infrared thermal camera within the first three minutes, as shown in Fig. 5a (see Supplementary Information S14 for details). We compare the temperature changes at the centre of the five samples before and after the experiment (cf. Fig. S14b), in which we can see that the Ni-LMMRE and Ni-MRE have the fastest temperature rise (~15 ℃ temperature change in 3 min) in comparison to other materials. We know that α can be determined as: $\alpha = k/\rho c_p$, where k is thermal conductivity, ρ is density, and c_p is specific heat capacity. Together, ρc_p is the volumetric heat capacity. We calculated the values of ρc_p for these five materials and the results are listed in Table S2 of the Supplementary Information S15. We found that the difference in ρc_p of the five samples is insignificant and therefore, their α are mainly determined by k. We can see that the PDMS-EGaIn composite has the lowest α, while the Ni-LMMRE and Ni-MRE have the highest α due to the irregular shape of Ni particles with a large k (90.7 W/m K). Compared with spherical iron particles, these irregular Ni particles with granular protrusions on the surface have more opportunities to contact with each other, and thus can significantly improve the thermal conductivity of the composite. Although the thermal conductivity of Ni-MRE is slightly higher, Ni-LMMRE has much lower Young's modulus and resistivity, which provides advantages for its application in flexible heating devices.

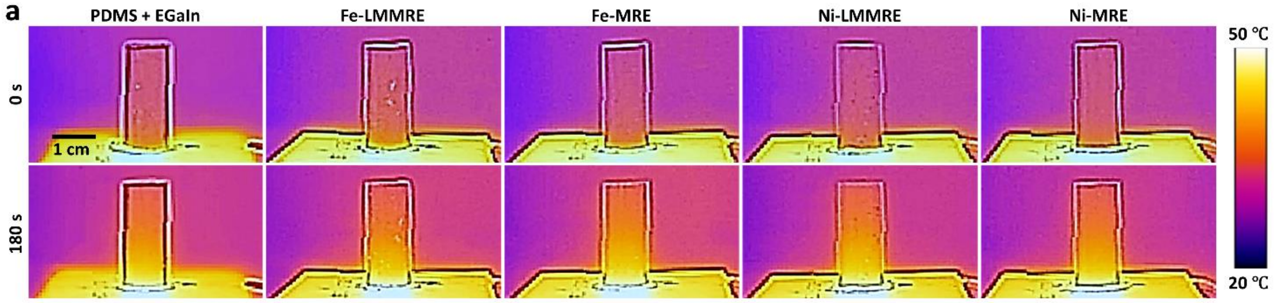

Fig. 5 a Thermal images of the cylindrical PDMS-EGaIn composite, Fe-/Ni-MRE, and Fe-/Ni-LMMRE on a thermoelectric plate.

Supplementary Information S14

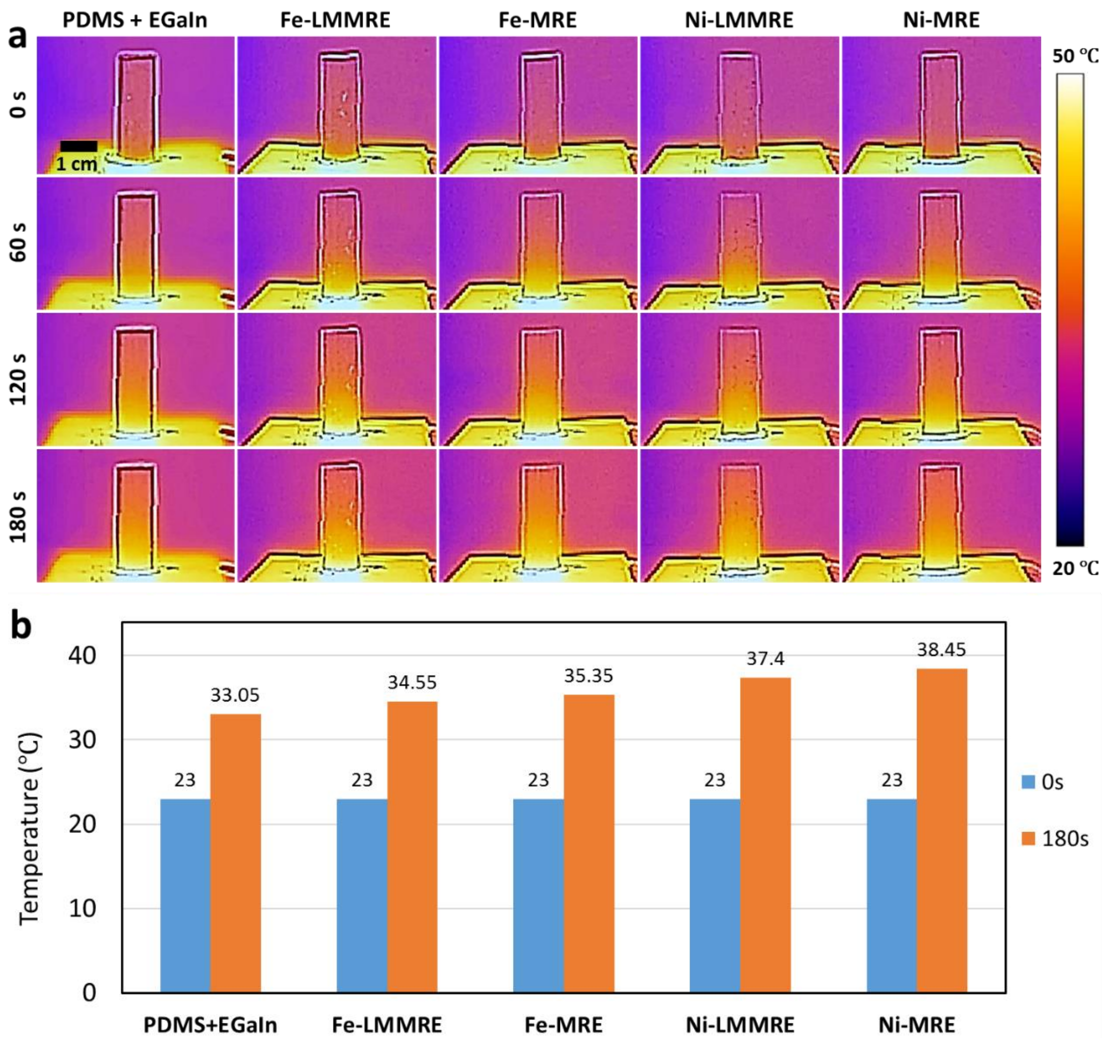

Figure S14. Thermal conduction experiment of the LMMRE. a Thermal images of the cylindrical PDMS-EGaIn composite, Fe-/Ni-MRE, and Fe-/Ni-LMMRE on a thermoelectric plate. **b** Temperature change at the centre of the five samples before and after the experiment.

In the thermal conduction experiment, the initial temperature of the five cylindrical samples was 23 ℃. Figure S14a shows the thermal images of these samples during the experiment. The temperature at the centre of these samples at 0 and 180 seconds were recorded as shown in Fig. S14b.

Supplementary Information S15: Calculation of the volumetric heat capacity for the composites

The volumetric heat capacity (ρc_p) is the product of the material density (ρ) and the specific heat capacity (c_p). It describes the ability of a unit volume of a material to store internal energy when undergoing a given temperature change (without undergoing a phase transition). To calculate the volumetric heat capacity, we need to know the density and specific heat capacity of each material. Density can be obtained by weighing the mass of a known volume of material, as given in Table S2. The specific heat capacity of a composite is equal to the mass-weighted average of the specific heat

capacities of the individual components, which can be calculated as below:

$$c = \frac{\sum_{i=1}^{n} m_i c_i}{\sum_{i=1}^{n} m_i}$$

The specific heat capacities of PDMS, Nickel, Iron, Gallium and Indium at room temperature are 1.46, 0.44, 0.44, 0.37 and 0.23 J/g K, respectively. According to this formula and the mass ratio of each component in the five materials, we can calculate their specific heat capacity and volumetric heat capacity as given in Table S2.

Table S2. Density, thermal conductivity and volumetric heat capacity of five materials.

	**PDMS-EGaIn**	**Fe-LMMRE**	**Fe-MRE**	**Ni-LMMRE**	**Ni-MRE**
ρ (g/cm^3)	2.608	2.885	3.105	2.965	3.269
c_p (J/(g K))	0.623	0.645	0.660	0.633	0.639
ρc_p (J/(cm^{3}K))	1.625	1.861	2.049	1.877	2.089

Although metal particles slightly increase the material density, the difference in the volumetric heat capacity of the five samples is insignificant. Therefore, their thermal diffusivity is mainly determined by their thermal conductivity *k*.

The *k* of EGaIn is only 26.6 W/m K, this makes the temperature of PDMS-EGaIn sample changed slowly. In addition, due to the lack of metal particle filler, the viscosity of the mixed liquid is low and thus does not shear the liquid metal significantly during stirring. As a result, the size of the EGaIn droplets is large (30-50 μm in diameter), as shown in the SEM image given in Fig. S15a. The PDMS between adjacent EGaIn droplets could be as thick as tens of microns, so the thermal conductivity of the PDMS-EGaIn composite is the smallest among the five materials.

The SEM images (Figs. S15b and 15c) show that the distribution of Fe and Ni particles in Fe- and Ni-MRE sample is quite dense, which in turn reduced the limitation of the PDMS on the heat transfer and improved the thermal conductivity. Figs. S15d and 15e show that Fe- and Ni-LMMRE have the similar microstructure. Since the difference of *k* between Fe (80.2 W/m K) and Ni (90.7 W/m K) is very small, we believe that the large difference in thermal conductivity between Fe-composites and Ni-composites is due to the irregular geometry of Ni particles. At the same concentration, the irregular Ni particles with granular protrusions on the surface have more opportunities to contact with each other than the spherical Fe particles, which in turn can significantly improve the thermal conductivity of the composite. Based on our experimental results and the above analysis, the Ni-LMMRE developed in this work has a high thermal diffusivity.

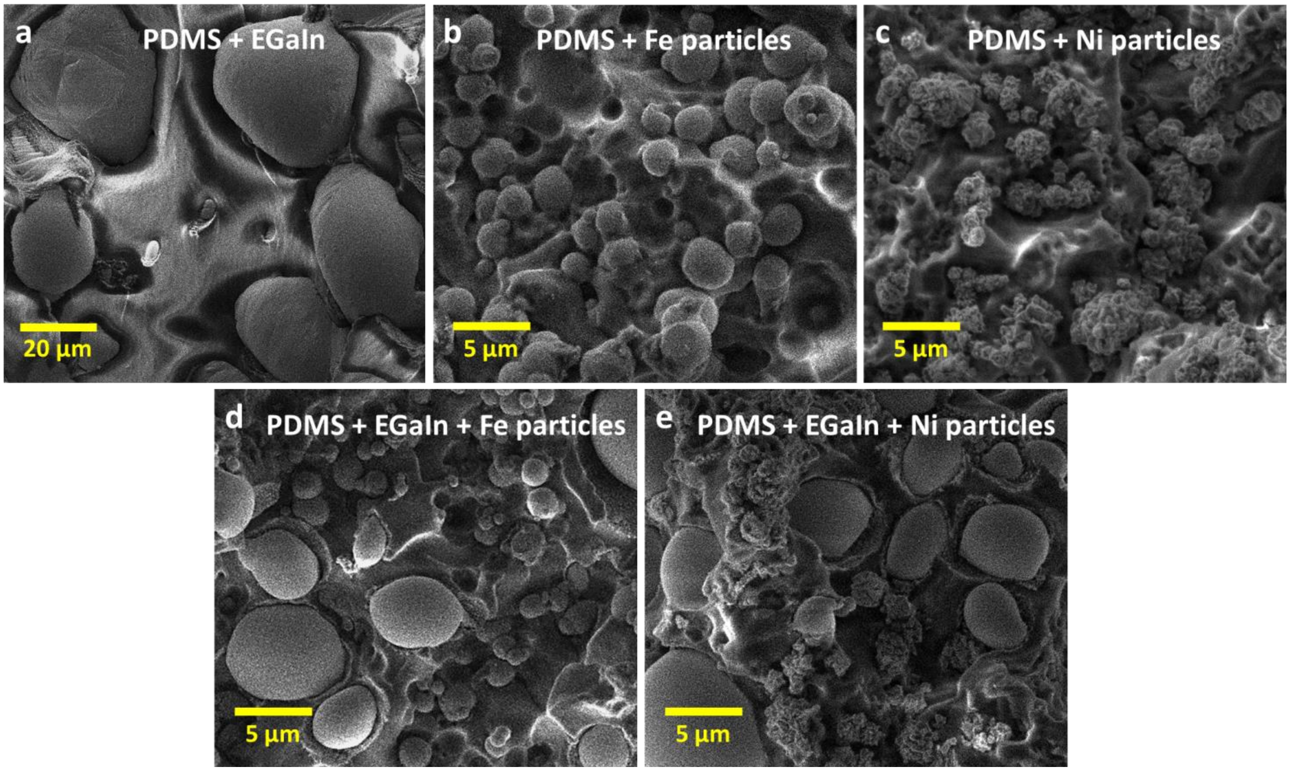

Figure S15. SEM images of the PDMS-EGaIn, Fe-MRE, Ni-MRE, Fe-LMMRE, and Ni-LMMRE.

6. The functionality presented in Figure 5b-g is quite interesting. Is this process reversible? Or does the copper foil plastically deform when pressure is applied? Cyclic compression experiments would be help to further understand this response.

Per the reviewer's comment, we conducted additional cyclic compression experiments on the heating devices presented in Figure 5. The experimental results and analysis are given in Supplementary Information S16:

Supplementary Information S16

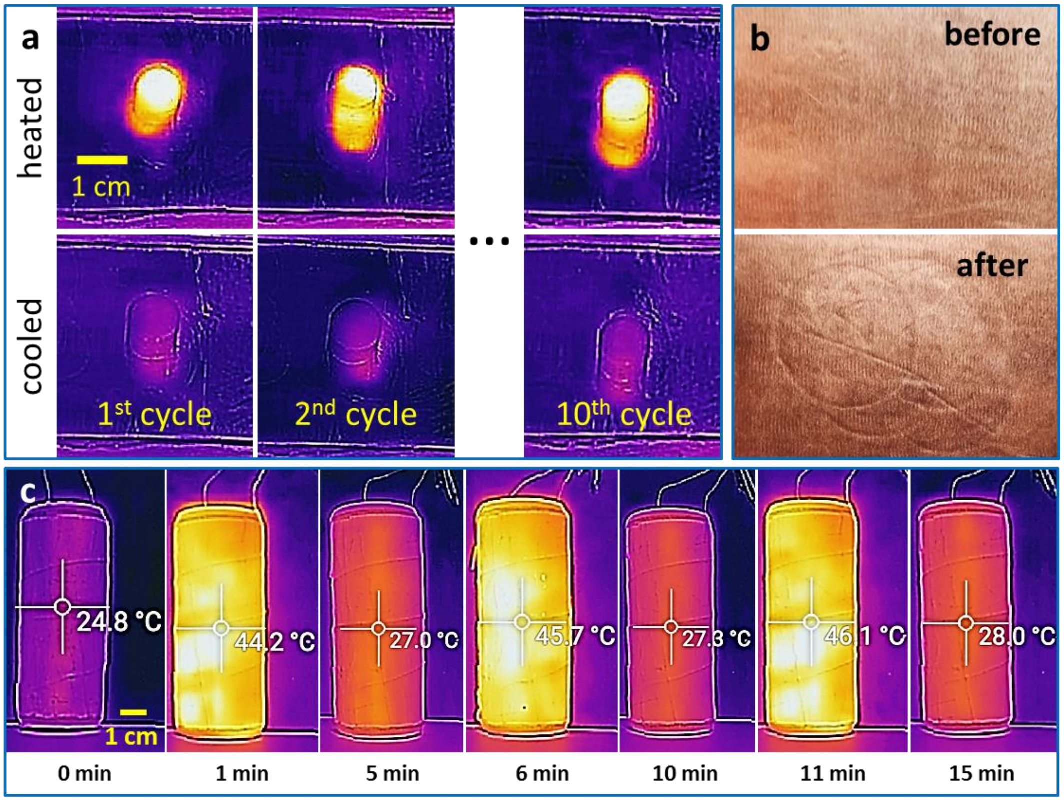

Figure S16. Cyclic compression experiments of the heating devices. Thermal images of the cycle compression experiments of **a** the pressure-sensitive heating device and **c** the hand-held heating column. **b** The photographs of the copper foil on the surface of the heated film before and after the cyclic compression experiment.

We conducted cyclic compression tests for the heating devices given in Figs. 5b and 5g. For the pressure-sensitive heating device in given Fig. 5b, we first placed a plastic cylinder (1 cm diameter) on the film and applied a pressure of about 400 kPa. The cylinder is heated to 40 ℃ after one minute. We then removed the pressure and cooled the heating device for one minute at room temperature (23 ℃). This process was repeated ten times, and the thermal images at the end of each heating and cooling is given in Fig. S16a. Fig. S16b shows the photographs of the copper foil on the surface of the heated film before and after the experiment. There were some slight indentations on the surface of the copper foil but the deformation is minimal. The copper foil did not undergo a severe plastic deformation since the strain of the film was small when activating the heating device (generally less than 5%). Once the external force is removed, the film can be restored to its original state immediately, and the copper foil will also be restored. Therefore, the slight deformation of the copper foil does not affect the repeated use of the heating device.

For the hand-held heating column presented in Fig. 5g, it was first gripped for one minute to generate heat, and then cooled at room temperature for four minutes. We repeated this process for three times. Thermal images of each cycle are shown in Fig. S16c. As we can see, the initial temperature of the heating column was 24.8 ℃. In each cycle, it can be heated to ~45 ℃ in one minute and cooled to below 30 ℃ after four minutes, indicating its ability for repeated work.

7. Overall, the paper is lacking significant details within the methods section. Currently there are no details about how the different composites are fabricated or the measurement techniques used for characterization. For example, as most multimeters cannot register resistances above 10 megaohms, how was the electrical conductivity of the composite measured?

Per the reviewer's comment, we added the following detailed information into the methods section. The multimeter we used (VC8145, VICI Digital Multimeter) is capable of measuring resistance up to 100 MΩ.

Page 26, Line 3

Methods

Materials and preparation of the conductive elastic composites:

EGaIn liquid metal and hydroxy iron powder were purchased from Sigma Aldrich, Australia. SYLGARD® 184 Silicone Elastomer Curing Agent and SYLGARD® 184 Silicone Elastomer Base were purchased from Dow Corning, American. The copper, nickel, zinc, silver, molybdenum and cobalt powder were purchased from NAIYATE Alloy welding material Ltd., China.

When preparing the sample, we first weighed the raw materials and placed them in a plastic test tube with a diameter of 15 mm in the order of PDMS-Fe powder-EGaIn, then stirred the mixture using a high-speed electric stirrer (rotating speed ranging from 400 to 2000 rpm) equipped with a plastic stick (diameter of 4 mm) for 5 min. After that, we vacuumed the mixture for 15 minutes to remove air bubbles and poured it into a mould made of poly(methyl methacrylate (PMMA) board. The mould was placed in the oven (70 ℃) for 6 hours to obtain the LMMRE.

Experimental equipment and tools：

A Screw Driven Linear Guide is used to form mechanical deformation of the LMMRE sample to measure its electrical characteristics. The two ends of the LMMRE sample were pasted with copper electrodes and fixed on the Linear Guide. In the compression and stretching test, the block sample (6 × 10 × 10 mm) and strip sample (4 × 6 × 10 mm) were uniformly compressed or stretched with a speed of 0.4 mm/min. In the bending test, the size of the strip sample was 3 × 6 × 40 mm. A VICI Digital Multimeter (VC8145) is used to measure electrical data such as the resistance of the composite and the current of the heating device. The digital multimeter has a resistance range of 100 MΩ, so it can measure a resistivity of one million Ω·m for the block sample (6 × 10 × 10 mm). The MTS Landmark 370.02 hydraulic load frame is used to measure the stress-strain curve and Young's modulus of the composites. SEM images were obtained using a JEOL JSM-6490LA scanning electron microscope. A Cat S60 FLIR infrared thermal camera was used to obtain the thermal images and videos. COMSOL Multiphysics 5.1 software package (Burlington, MA, USA) was used to simulate the distribution of Fe microparticles and EGaIn microdroplet upon stretching, as well as calculate the change of resistivity of the LMMRE.

Minor comments:

- Because of the large difference in density, it would be helpful to see the ratios of polymer and metal filler expressed as a volume fraction of the composite, in addition to mass fraction which is currently stated within the manuscript.

Per the reviewer's comment, we have indicated the volume fractions for all materials in the main manuscript.

- For all cyclic loading experiments, it would be helpful for the authors to present not only the last few loading cycles but also loading cycles at different points within the experiment: beginning, middle and end.

Per the reviewer's comment, we added the following figures and descriptions in Supplementary Information S8 to show the resistance change during the first few loading cycles. The resistance change for the last few cycles are given in Figure 2 in the main manuscript.

Supplementary Information S8

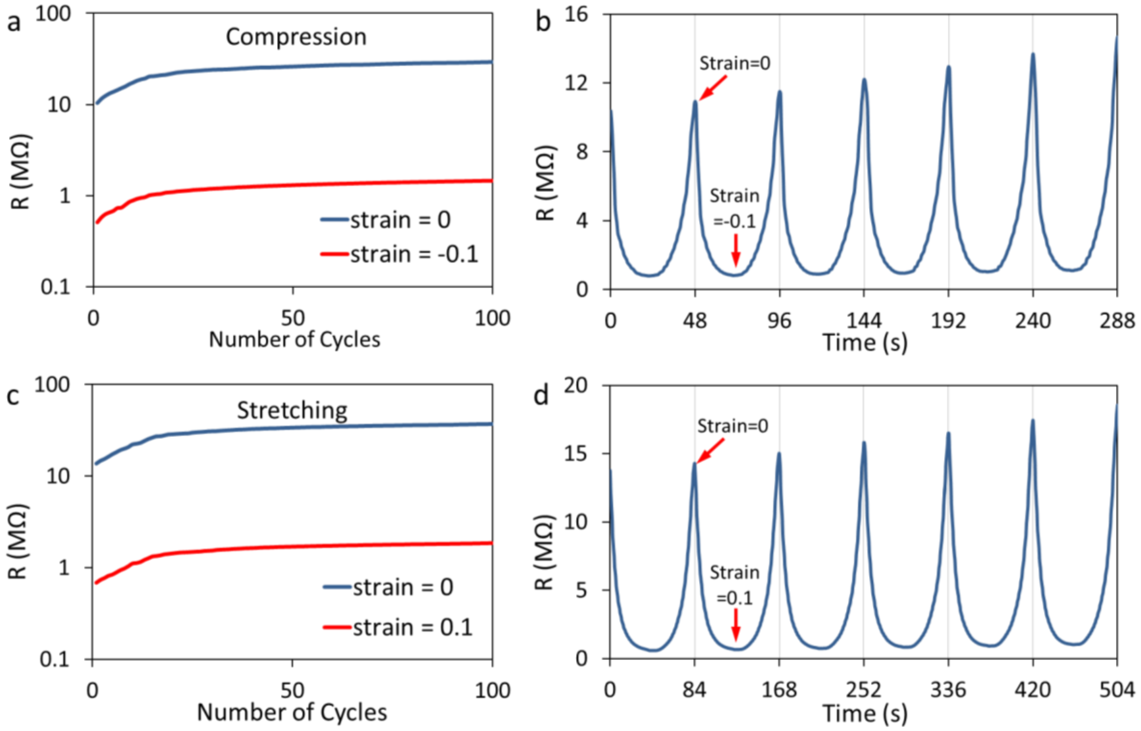

Figure S8. Change of resistance for the LMMRE under cyclic loading. Resistance changes of the LMMRE under **a** cyclic compression load and **c** cyclic tensile load. Resistance changes of the LMMRE during the first six cycles of **b** cyclic compression load and **d** cyclic tensile load.

In this cyclic experiment, the sample was uniformly compressed/stretched by 10% and restored to its original length >100 times. In the compression test, the sample had an initial resistance (strain = 0) of 10.4 MΩ. Then the resistance began to rise rapidly (Fig. S8b) and gradually stabilised after 15 cycles. At the end of the experiment, the resistances at the relaxed state and under a −0.1 compressive strain were

maintained at 30 and 1.5 MΩ, respectively. A similar phenomenon was observed in the tensile test. The sample resistivity in the relaxed state was stabilised after 15 cycles. The increase in resistivity during cyclic test could be attributed to the redistribution of EGaIn microdroplets within the LMMRE.

- Does curing temperature have an influence on the resistance of the composite?

Per the reviewer's comment, we examined the influence of curing temperature on the resistance of the LMMRE, as given in Fig. S6b of the Supplementary Information S8:

Supplementary Information S6: Additional variables that affect the resistivity of the LMMRE

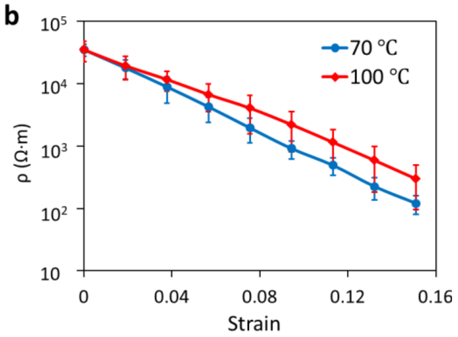

Figure S6b. Resistivity of the Fe-LMMRE prepared using different curing temperatures.

In addition to the materials used for preparing the LMMRE, we found that the curing temperature and time also affect the electrical properties of the material. It takes several days for the mixed liquid to solidify at room temperature, so it is necessary to heat it to speed up the process. To determine the appropriate heating temperature, we cured two samples at 70 and 100 ℃ for 6 h, respectively, and obtained their resistivity-strain curves (Fig. S6b). Although their resistivity is similar in the relaxed state, the higher curing temperature may slightly weaken its sensitivity to compression. So we set the heating temperature at 70 ℃.

- Typo: 30 V voltage (page 20)

Per the reviewer's comments, we fixed the mistake.

- Can the authors expand upon this statement (page 20, first paragraph): "In addition, the simple circuit structure makes it less susceptible to damage." It is currently unclear to the reader how this circuit structure is less susceptible to damage or what this circuit should be compared to.

Per the reviewer's comments, we revised our descriptions to avoid confusion, as given below:

Page 22, Line 22

In addition, the device has no complicated circuit structure or uses any pressure sensor, which reduces the risk of damage and makes it easy to repair.

The original intention of this sentence is to express that the structure of this heating device is very simple. Although it can automatically adjust the temperature according to the pressure, it does not need

a pressure sensor. Essentially, it is just a heating resistor powered by a DC source. Therefore, it is less susceptible to damage than devices that require a pressure sensor to control the circuit.
- Page 23: "The LMMRE can therefore have both the high gauge factor and the low electrical resistance at *high* strains." Currently, all demonstrations are shown at fairly low strains < 20%
Per the reviewer's comments, we revised our descriptions to clarify the confusion, as given below:<br>***Page 25, Line 10***<br>The LMMRE can therefore have both a high piezoconductive coefficient and a low electrical resistance during elongation.
- In Figure 4b, what does the initial rise in resistance from approximately 0 to 10 seconds correspond to?
The LMMRE sample was compressed by 5% at 0 s, and then placed in a uniform magnetic field for the cycling test. After compression, the microstructure of the elastomer has a tendency to recover. Therefore, its resistance will slowly rise and stabilise within around 30 seconds. That is why the resistance increased from 0 to 11 seconds and 15 to 25 seconds in Figure 4b. After 30 seconds, the resistance of LMMRE was basically stable and did not affect the cycling test in the magnetic field. This is also why we waited until 40 seconds to start the cycling test.
- "As a control, we fabricated a composite only containing PDMS (1 g) and Ni particles (2 g) without using EGaIn, and found that this composite is basically an insulator under compressive strains small than –0.05." What does basically an insulator refer to?
In this work, we define a composite as an insulator when its resistivity exceeds 1 MΩ·m. We added the following descriptions in the main manuscript to avoid confusion:<br>***Page 16, Line 9***<br>As a control, we fabricated a composite only containing PDMS (1 g, volume fraction of 81.6%) and Ni particles (2 g, volume fraction of 18.4%) without using EGaIn, and found that this composite is effectively an insulator (resistivity exceeds 10^6 Ω·m) under compressive strains small than –0.05.
- Page 20: "Due to the positive piezoconductive characteristic of the LMMRE, this heating film can also generate heat during stretching when applying a current." The composite cannot generate heat but is capable of *producing* heat based on the Joule heating effect.
Per the reviewer's comments, we revised our descriptions to clarify the confusion, as given below:<br>***Page 23, Line 3***<br>Due to the positive piezoconductive characteristic of the LMMRE, this heating film can also produce heat during stretching based on the Joule heating effect when applying a voltage.

REVIEWERS' COMMENTS:

Reviewer #1 (Remarks to the Author):

The authors answered convincingly to the Referees' comments. I think the manuscript can be published in Nature Communications.

Reviewer #2 (Remarks to the Author):

The authors have addressed the comments raised by the reviewers and have revised the manuscript accordingly. It should be accepted for publication.
